# Using mHealth Technologies for Case Finding in Tuberculosis and Other Infectious Diseases in Africa: Systematic Review

**DOI:** 10.2196/53211

**Published:** 2024-08-26

**Authors:** Don Lawrence Mudzengi, Herbert Chomutare, Jeniffer Nagudi, Thobani Ntshiqa, J Lucian Davis, Salome Charalambous, Kavindhran Velen

**Affiliations:** 1 The Aurum Institute Johannesburg South Africa; 2 School of Public Health University of the Witwatersrand Johannesburg South Africa; 3 Ministry of Health LISTEN Project Mbabane Swaziland; 4 Department of Epidemiology of Microbial Diseases Yale School of Public Health Connecticut, CT United States

**Keywords:** mobile health, mHealth, design thinking, tuberculosis, Ebola, HIV, COVID-19, infectious diseases, contact tracing, mobile phone

## Abstract

**Background:**

Mobile health (mHealth) technologies are increasingly used in contact tracing and case finding, enhancing and replacing traditional methods for managing infectious diseases such as Ebola, tuberculosis, COVID-19, and HIV. However, the variations in their development approaches, implementation scopes, and effectiveness introduce uncertainty regarding their potential to improve public health outcomes.

**Objective:**

We conducted this systematic review to explore how mHealth technologies are developed, implemented, and evaluated. We aimed to deepen our understanding of mHealth’s role in contact tracing, enhancing both the implementation and overall health outcomes.

**Methods:**

We searched and reviewed studies conducted in Africa focusing on tuberculosis, Ebola, HIV, and COVID-19 and published between 1990 and 2023 using the PubMed, Scopus, Web of Science, and Google Scholar databases. We followed the PRISMA (Preferred Reporting Items for Systematic Reviews and Meta-Analyses) guidelines to review, synthesize, and report the findings from articles that met our criteria.

**Results:**

We identified 11,943 articles, but only 19 (0.16%) met our criteria, revealing a large gap in technologies specifically aimed at case finding and contact tracing of infectious diseases. These technologies addressed a broad spectrum of diseases, with a predominant focus on Ebola and tuberculosis. The type of technologies used ranged from mobile data collection platforms and smartphone apps to advanced geographic information systems (GISs) and bidirectional communication systems. Technologies deployed in programmatic settings, often developed using design thinking frameworks, were backed by significant funding and often deployed at a large scale but frequently lacked rigorous evaluations. In contrast, technologies used in research settings, although providing more detailed evaluation of both technical performance and health outcomes, were constrained by scale and insufficient funding. These challenges not only prevented these technologies from being tested on a wider scale but also hindered their ability to provide actionable and generalizable insights that could inform public health policies effectively.

**Conclusions:**

Overall, this review underscored a need for organized development approaches and comprehensive evaluations. A significant gap exists between the expansive deployment of mHealth technologies in programmatic settings, which are typically well funded and rigorously developed, and the more robust evaluations necessary to ascertain their effectiveness. Future research should consider integrating the robust evaluations often found in research settings with the scale and developmental rigor of programmatic implementations. By embedding advanced research methodologies within programmatic frameworks at the design thinking stage, mHealth technologies can potentially become technically viable and effectively meet specific contact tracing health outcomes to inform policy effectively.

## Introduction

### Background

Mobile health (mHealth) technologies have become increasingly popular tools for facilitating data collection and delivery of health services worldwide [1,2]. The emergence of COVID-19 in 2020 increased interest in using mHealth technologies for contact tracing due to their ability to achieve case finding goals without the need for physical contact with an infected person [1,2]. Before COVID-19, mHealth technologies had already been used in Africa for contact tracing of infectious diseases such as Ebola [3-7], tuberculosis [8-11], and HIV, albeit with limited success. Compared to paper-based systems, the most apparent advantages of mHealth include its ability to reduce repetitive tasks and errors, systematic delivery of services, and improved monitoring due to efficient data processing in databases. Therefore, at face value, mHealth technologies have the potential to overcome challenges encountered when using traditional paper-based systems for contact tracing, thereby improving outcomes.

Contact tracing is a strategy for actively and systematically screening for symptoms among individuals exposed to someone with a transmissible disease to determine whether they require further diagnostic evaluation [12-14]. A key advantage of contact tracing is that, in principle, it reduces the time from when an individual falls ill with an infectious disease to when they are diagnosed, preventing further transmission to healthy persons. A contact, defined as any person living with or someone in a social circle who has regular contact with an individual with a transmissible disease, is at the highest risk of contracting the same disease due to their proximity to the infected person. Therefore, contacts are defined as a high-risk priority group for contact tracing [12]. The World Health Organization has long recommended and supported contact tracing for tuberculosis and Ebola [15,16] and promotes contact tracing as a critical intervention for tuberculosis control in high-burden countries [16].

Tuberculosis contact tracing can be seen as a cascade of activities that begins with finding an individual with the disease of interest, referred to as the index patient, and collecting information about their close contacts [12]. These activities are followed by a household visit to screen the enumerated persons for tuberculosis symptoms and may include collecting sputum samples from symptomatic contacts for laboratory testing—anyone testing positive for tuberculosis is referred to health facilities for linkage to care [12]. During household visits, contact tracing also serves as a pathway for accessing household contacts eligible for and initiating tuberculosis preventive therapy [14]. There are variations to contact tracing, with recent modalities using portable chest x-rays for identifying individuals eligible for tuberculosis testing [17,18] and oral swabs as an alternative to sputum samples [19,20]. In South Africa, contact tracing has now evolved from testing only symptomatic persons to universal testing of all household contacts and, in the process, initiating tuberculosis preventive therapy in eligible contacts [21].

The process for conducting contact tracing is rigorous. Each step in the contact tracing cascade requires documentation to enable contact follow-up, communication of results, linkage to care, and program monitoring and evaluation. Contact tracing programs rely significantly on efficient data collection to inform decision-making and patient management—in the absence of this, the process loses its cost-effectiveness and may become unattractive to national tuberculosis programs. Within Africa, countries have relied on inefficient paper-based data collection that overburdens outreach workers responsible for tracing household contacts but also results in poor data quality due to inevitable human error and inadequate accountability due to the manual processes required to collate field data [12,22-25]. These systems could be improved or overhauled by introducing mHealth technologies to optimize the documentation of activities in each step of the cascade.

### Objectives

Despite the potential of mHealth to overcome traditional paper-based system challenges, there is evidence suggesting that improvements in contact tracing outcomes using mHealth technologies remain insufficient and “largely unproven” [24,26-28]. To address the evidence gaps, this systematic review assessed technologies on tuberculosis, Ebola, HIV, and COVID-19. While our focus remains predominantly on tuberculosis, we included these additional diseases due to similarities in contact tracing methods, enhancing the wider applicability of our findings. We aimed to synthesize information on the development, implementation, and evaluation of these technologies to better guide future work and enhancements. We hypothesized that synthesizing this existing information on the technologies will reveal valuable insights into the continuum of mHealth apps, their benefits, and limitations, thereby shaping improvements in future contact tracing efforts.

## Methods

### Literature Search

We conducted a systematic review of mHealth technologies used for contact tracing for selected infectious diseases and reported the results using the PRISMA (Preferred Reporting Items for Systematic Reviews and Meta-Analyses) guidelines. We conducted an iterative search of all studies published in or translated into English from January 1, 1990, to December 31, 2023, in electronic databases, including PubMed (MEDLINE), Scopus, Web of Science, and Google Scholar (Multimedia Appendix 1). The year 1990 was chosen because it represents the earliest period during which the literature suggests that digital phones were introduced and available for use in health [29,30]. In addition, the 2023 cutoff allowed for the inclusion of technologies that may have been used during the COVID-19 pandemic. The following search terms were applied within the selected databases: *tuberculosis*, *mHealth* or *mobile health*, *telehealth* or *telemedicine* and *case finding* or *contact tracing*/*investigation* or *tuberculosis screening* or *COVID-19* or *Ebola* or *HIV*. We used Medical Subject Heading (MeSH) terms to search in PubMed. We used subject headings and keywords in databases that do not use MeSH terms, such as Scopus, Web of Science, and Google Scholar. The search terms are presented in Multimedia Appendix 1. The Boolean operators “AND,” “OR,” and “NOT” were used to join words in all databases to improve the accuracy and relevance of the literature.

### Inclusion and Exclusion Criteria

The following study inclusion criteria were applied: (1) studies conducted in Africa and published between 1990 and 2023 (2) that included an mHealth technology either as an intervention or part of procedures for contact tracing (3) used to screen for tuberculosis, COVID-19, Ebola, and HIV or find and screen contacts of people infected with these diseases.

The following studies were excluded: (1) mHealth modeling studies; (2) mHealth protocols and proposals; and (3) mHealth systematic reviews, commentaries, and scoping reviews.

### Screening of the Literature, Extraction, and Analysis

The initial database search was conducted by DLM, and all references were uploaded to a reference management software library (EndNote version 20; Clarivate Analytics) for abstract and title screening. In total, 2 reviewers (DLM and HC) performed the initial review of all articles according to the inclusion and exclusion criteria. Where there was disagreement on the classification of a reference, a third reviewer (JN) conducted an additional review and confirmed the final classification.

A web-based data extraction tool was developed on Microsoft Office 365 Forms (Microsoft Corp; Multimedia Appendix 2). Extracted data elements included the study title, year of publication, country, location where the technology was implemented (community, facility, or both), study design, target disease, and type of technology used. The same form also contained sections to capture qualitative data about how the technologies were developed and their implementation processes, including challenges and the outcomes to measure effectiveness.

We summarized and synthesized the systematic review results using the Joanna Briggs Institute approach [31], and the data were presented in tables and narrative text. The themes used in the analysis were predetermined: development, implementation, and outcomes. The development theme described the steps taken in the development of the technologies. The implementation theme described how the technologies were deployed, what and how they collected contact tracing data, and challenges and successes. The final theme focused on the contact tracing outcomes measured when mHealth technologies were used.

### Quality Assessments and Risk of Bias

The included studies were assessed for risk of bias using tools appropriate for each study type. The steps followed in assessing risk are detailed in Multimedia Appendix 3 [6,8,27,32-48]. The Cochrane Risk of Bias 2 tool was used for randomized clinical trials [49]. Technologies used in programmatic settings and pretest-posttest studies were assessed using the Quality Assessment Tool for Before-After (pretest-posttest) Studies With No Control Group developed by the US National Heart, Lung, and Blood Institute [50]. Finally, cross-sectional studies were assessed using the Quality Assessment Tool for Observational Cohort and Cross-Sectional Studies also from the National Heart, Lung, and Blood Institute [50].

### Definitions

The World Health Organization broadly defines eHealth as “the use of information and communications technology (ICT) in support of health and health-related fields, including health care services, health surveillance, health literature, health education, knowledge and research” [51].

mHealth is a branch of eHealth that refers to mobile wireless technologies to support public health objectives [51,52]. mHealth technologies are mainly used on portable devices such as phones and tablets [52,53] and allow for the ubiquitous provision of services such as public health surveillance, sharing of clinical information, data collection, health behavior communication, and the use of mobile technologies to reach health goals [54].

“Programmatic pretest-posttest” describes the use of technologies in programmatic settings to deliver an interventions to large populations targeting specific outputs and outcomes.

“In-house software platform” refers to bespoke technologies without using existing platforms. For example, many public health projects build forms on platforms such as Open Data Kit (ODK), Epi Info, and REDCap (Research Electronic Data Capture; Vanderbilt University), and in this review, these were not considered in-house software platforms.

“The effectiveness of an mHealth technology used in programmatic settings” is defined in the context of a project or program designed to implement an intervention with a target and without the use of research principles. Effectiveness in this sense is the achievement of a set target, such as the number of people screened using an mHealth technology.

“Effectiveness in a research project of an mHealth technology” is defined as the improvement of a contact tracing outcome before and after, such as the number of people diagnosed when screened using an mHealth technology.

## Results

### Literature Search

**Figure 1:**
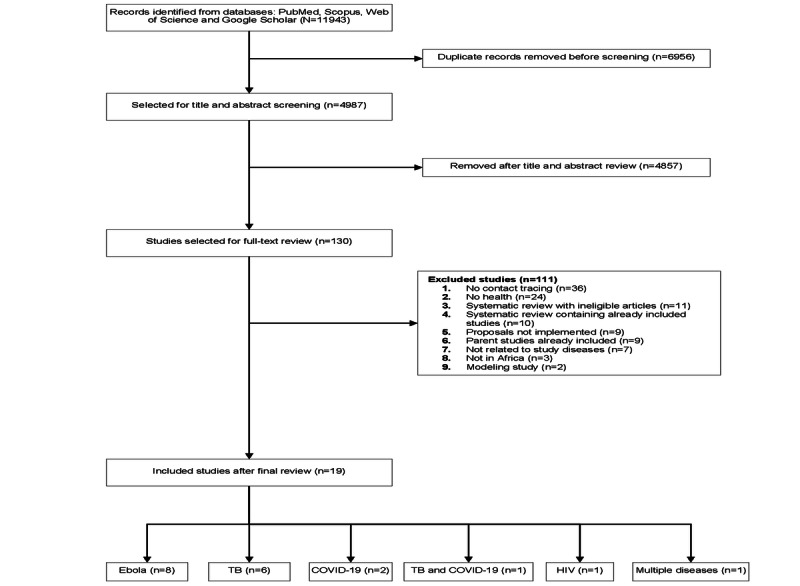
The PRISMA (Preferred Reporting Items for Systematic Reviews and Meta-Analyses) flow diagram for the systematic review. TB: tuberculosis.

Figure 1 provides a PRISMA flowchart for the literature search and review process. The initial literature search yielded 11,943 articles. After removing 58.24% (6956/11,943) of duplicates, 41.76% (4987/11,943) of the articles remained for screening. A review of titles and abstracts led to the exclusion of 97.39% (4857/4987) of the articles, resulting in 130 articles being selected for full-text review. Only 19 studies were retained after the full-text review [6,8,27,32-47]. Of these 19 studies, 8 (42%) were on Ebola [32-39], 6 (32%) were on tuberculosis [6,27,40-43], 2 (11%) were on COVID-19 [44,45], 1 (5%) was on HIV [8], 1 (5%) was on tuberculosis and COVID-19 [46], and 1 (5%) was on multiple diseases in humans and animals [47]. No study needed translation.

### Description of the Studies

Table 1 provides an overview of the key characteristics of the 19 studies that formed the basis of this systematic review. The studies covered a range of diseases, including Ebola, tuberculosis, COVID-19, HIV, and multiple diseases in humans and animals. In total, 74% (14/19) of the included studies were about technologies used for contact tracing of Ebola and tuberculosis. Ebola had the most studies with 42% (8/19), whereas tuberculosis had 32% (6/19). One other technology was used for both tuberculosis and COVID-19. The remaining 4 studies discussed technologies for the contact tracing of HIV (n=1, 25%), COVID-19 (n=2, 50%), and multiple diseases among humans and animals (n=1, 25%). Multimedia Appendix 4 [6,8,27,32-47,55-75] provides a detailed summary of each technology.

**Table 1:** Description of all retained studies.

Disease and target users		Technology type	Study	Country	Study design	Location
**Ebola**						
	Health workers	Smartphone app, GIS^a^, and BI^b^ dashboard	Tom-Aba et al [34], 2015	Nigeria	Programmatic pretest-posttest	Community
	Health workers	Smartphone app and BI dashboard	Sacks et al [32], 2015	Guinea	Programmatic pretest-posttest	Community
	Contacts	SMS text messaging and phone calls	Jia and Mohamed [33], 2015	Sierra Leone	Programmatic pretest-posttest	Community
	Health workers	Smartphone app	Adeoye et al [37], 2017	Nigeria	Programmatic pretest-posttest	Community
	Contacts	Phone calls	Alpren et al [36], 2017	Sierra Leone	Programmatic pretest-posttest	Community
	Health workers	Mobile tracking	Wolfe et al [35], 2017	Liberia	Programmatic pretest-posttest	Community
	Health workers	Smartphone app	Danquah et al [38], 2019	Sierra Leone	Cross-sectional	Community
	Health workers	Smartphone app	Whitesell et al [39], 2021	DRC^c^	Programmatic pretest-posttest	Community
**Tuberculosis**						
	Health workers	Smartphone app and GIS	Chisunkha et al [6], 2016	Malawi	Cross-sectional	Community
	Health workers	Smartphone app	Ha et al [40], 2016	Botswana	Pretest-posttest	Community
	Contacts	USSD^d^	Diaz and Moturi [42], 2019	Tanzania	Programmatic pretest-posttest	Community
	Health workers	Smartphone app	Davis et al [27], 2019	Uganda	Trials	Used or intended for use in both the facility and community
	Health workers	Smartphone app	Szkwarko et al [41], 2021	Kenya	Cross-sectional	Facility
	Both	Smartphone app	URC^e^ [43], 2020	South Africa	Programmatic pretest-posttest	Used or intended for use in both the facility and community
**COVID-19**						
	Contacts	Web application and USSD	Owoyemi et al [45], 2021	Nigeria	Programmatic pretest-posttest	Community
	Contacts	Smartphone app	Mugenyi et al [44], 2021	Uganda	Pretest-posttest	Community
**Tuberculosis and COVID-19**						
	Contacts	USSD and WhatsApp	Praekelt.org [46], 2021	South Africa	Programmatic pretest-posttest	Community
**HIV**						
	Health workers	Smartphone app	Rajput et al [8], 2012	Kenya	Programmatic pretest-posttest	Community
**Multiple diseases**						
	Health workers	Smartphone app	Karimuribo et al [47], 2017	Tanzania	Programmatic pretest-posttest	Community

^a^GIS: geographic information system.

^b^BI: business intelligence.

^c^DRC: Democratic Republic of the Congo.

^d^USSD: unstructured supplementary service data.

^e^URC: University Research Co.

The type of mHealth technology varied across the selected studies. Exactly 42% (5/19) used communications platforms with SMS and phone calls accounting for 11% (2/19) [33,36] and unstructured supplementary service data or WhatsApp used in 16% (3/19) [42,45,46]. Smartphone apps were the most common, appearing in 68% (13/19) of the studies [6,8,27,32,34,37-41,43,44,47]. Among these smartphone apps, 25% (3/12) incorporated geographic information systems [6,34] or a business intelligence dashboard [32,34]. Only one out of the 19 (5%) used a mobile tracking system managed through cellphone tower technologies [35].

Most of the technologies (16/19, 84%) [6,8,32-40,42,44-47] were designed for use in the community during outreach visits, whereas 11% (2/19) were used in both the community [27,43] and the household and only 5% (1/19) were designed for use in a health facility [41]. Of all 19 technologies, 11 (58%) were designed for use by outreach workers to capture data during contact tracing [6,8,27,32,34,37-41,43,47], whereas 7 (37%) were designed for self-screening disease symptoms by the contacts [33,35,36,42,44-46] and only 1 (5%) could be used by both patients and outreach workers [43]. In contact-targeted technologies, contacts interacted with the system by sending messages, responding to built-in prompts, and making or receiving phone calls for contact tracing, whereas outreach workers used the technologies to facilitate data collection. Only 5% (1/19) of the studies used a technology to facilitate linkage to care of household contacts, a distal outcome from contact tracing [27].

Regarding study designs, 68% (13/19) of the studies that implemented technologies in programmatic settings used pretest-posttest designs. In these cases, the technology served as an intervention aimed at achieving specific contact tracing or case finding objectives within broad population programs, often without the technology of stringent research methodologies. The remaining 6 studies used various designs: 3 (50%) were pretest-posttest studies, 2 (33%) were cross-sectional studies, and 1 (17%) was a randomized trial.

### The Uses of Technologies in the Included Studies

The uses of the technologies in the included studies are also presented in Table 1. As predetermined in the inclusion criteria, all technologies were designed to assist in contact tracing and infectious disease case finding. However, some were primarily designed for disease surveillance incorporating contact-tracing functionality [41,45,47,76]. The predominant contact tracing modality in the technologies involved identifying index patients, finding their contacts, and collecting data using mobile technologies instead of paper.

Other studies used unconventional contact tracing methods that did not necessarily require initial contact with index patients or arrangements with household contacts. For instance, in a study conducted in Malawi by Chisunkha et al [6], Google Earth, a publicly available software, was used to identify and visit households for recruitment into a chronic airway disease and tuberculosis trial even in the absence of previous knowledge of index patients. This approach involved the use of global positioning technology to locate households within a disease hot spot area on a map. Subsequently, data collectors were dispatched to these identified locations to recruit participants for the trial. While the system was not exclusively designed as a contact tracing project but rather as a tool to facilitate the location of participants within a study, the authors proposed that it could also serve contact tracing purposes in remote settings. This approach allowed for the swift identification of households before actual visits, thereby reducing costs and enhancing efficiency [6]. In Monrovia, Liberia, Wolfe et al [35] described an Ebola contact tracing system in which the government issued subpoenas to cell phone companies forcing them to provide locations of contacts that outreach workers could not locate. The authors reported that these subpoenas assisted in successfully locating 29 missing contacts [35]. In Sierra Leone, Alpren et al [36] described a repurposed call center, also for Ebola, in which the public acted as primary informants to authorities to report details and locations of known or suspected patients with Ebola (live alerts) and deaths (death alerts). The study showed >10,000 weekly alerts at the peak of the Ebola outbreak in October 2014. Cumulatively, between 2014 and 2016, the call center received 248,789 death alerts and 95,136 live alerts [36]. Finally, Mugenyi et al [44] developed and implemented the Wetaase app in a small pilot study to monitor the symptoms and movement of household members during the COVID-19 outbreak in Uganda. Unique to this technology was that household members downloaded and self-reported daily symptoms through Wetaase, and if symptomatic, they were tested for COVID-19 by workers visiting the household. Only 101 participants were enrolled, and out of an expected daily report of 8949 in 90 days, the app achieved 6617 reports, a use rate of 78%. Of these 6617 reports, only 57 (0.8%) self-reported COVID-19 symptoms, and no cases were diagnosed.

### Development of mHealth Technologies for Contact Tracing

#### Type of Platform for Development

In total, 84% (16/19) of the technologies in the included studies were digital technologies developed to work on mobile phones. Of these 16 technologies, 11 (69%) were developed by customizing existing platforms such as ODK and CommCare, while 6 (38%) were developed as in-house software platforms. Of the 11 customized technologies, ODK was used to develop 5 (45%), and CommCare was used to develop 3 (27%) [27,32,38], while Unstructured Supplementary Service Data was used to develop 2 (18%) technologies and only 1 (9%) was developed using KoboToolbox. The remaining 16% (3/19) of the technologies used existing cellular network infrastructure such as SMS text messaging and phone calls. Of these 3 technologies that used existing cellular network infrastructure, 2 (67%) used phone calls, while 1 (33%) used cell phone tower technology.

#### Scope and Scale of Implementation

The scope and implementation scale of the technologies in the included studies varied depending on the intended use, that is, in programmatic or research settings. Technologies used in programmatic settings of contact tracing activities had a larger scope aimed at targeting large sections of the population to meet contact tracing targets of the program. Large-scope technologies in the selected studies included the Surveillance Outbreak Response Management and Analysis System (SORMAS) in Nigeria, the Academic Model Providing Access to Healthcare (AMPATH) in Kenya, Tanzania’s Tambua tuberculosis and Afyadata, tuberculosis HealthCheck in South Africa, and the Ebola contact tracing app in Guinea. For example, AMPATH in Kenya was developed to facilitate screening among >2 million individuals in 3 years [8]. Similarly, HealthCheck in South Africa was initially designed for population-wide self-screening of COVID-19 and expanded to tuberculosis at a national level [46]. In Tanzania, Afyadata, which veterinary specialists conceptualized, had a large scope for screening and reporting diseases in animals and humans [47,55]. Among humans, Afyadata has been used for finding diseases such as Ebola [47,77,78], COVID-19 [56,79], cholera [55,80,81], brucellosis [82,83], and an impetigo-like outbreak [47,55,84]. In contrast, technologies developed for research studies had a narrow scope, mainly built to demonstrate the value of mHealth in research settings or as research data collection tools, and had no evidence of uptake beyond the research. In Botswana, Ha et al [40] demonstrated that mHealth was better than paper by developing a mobile technology using ODK, but only 376 contacts were screened before the technology was probably retired. Chisunkha et al [6] developed a mobile technology using ODK to enumerate households for a tuberculosis contact tracing trial in Malawi and a small population. We did not find any further information about the tenure of this mHealth technology after the enumeration of the households. In Kenya, Szkwarko et al [41] developed PPTBMAPP to facilitate tuberculosis screening of 276 children in a health facility and demonstrated its superiority over paper, but no further use is documented. Also in Uganda, a randomized trial used an mHealth technology developed using CommCare as a data collection and screening tool and then evaluated the effectiveness of SMS text messaging to complete tuberculosis evaluation within 2 weeks. This technology screened only 919 contacts in the intervention and control arms [27].

#### Funding for Developing mHealth Technologies

Large-scale technologies developed for programmatic settings often received substantial funding for extensive development and implementation processes. For instance, SORMAS received €850,000 (US $929,509) from the European Union [85], Afyadata obtained US $450,000 from the Skoll Global Threats Fund [55], and the CommCare app for HIV screening was supported by a US $74.9–million US Agency for International Development–AMPATH program [86]. Tambua tuberculosis received government backing through Tanzania’s National Tuberculosis and Leprosy Programme, which facilitated support and acceptance from end users [42].

In contrast, many research projects included in this review did not report their funding sources. This lack of reported funding highlights the resource disparities between technologies used in programmatic settings and research studies. Research projects often operated with limited financial resources, which impacted the scope and scale of their technology development processes. For example, in Botswana, Ha et al [40] evaluated a pragmatic mHealth technology for tuberculosis contact tracing. While the technology demonstrated promising outcomes compared to traditional paper-based methods, its application was limited to a pilot study across 7 urban and semiurban health facilities, and there is no record of its broader implementation, underscoring a challenge of limited scope in assessing the technology’s full potential and scalability [40].

#### Participatory Development Processes as an Indicator of Successful Implementation

In total, 21% (4/19) of the technologies—SORMAS [57,76], Afyadata [47,55], AMPATH [8], and Tambua tuberculosis [42]—documented the use of design thinking frameworks in their development processes. Design thinking frameworks increase the likelihood of producing technologies that are fit for the intended purposes by using participatory processes to capture all the requirements and what outcomes the technology must deliver and how and also address the context in which the technology will be used [57,58]. Design thinking also involves extensive consultation with relevant stakeholders such as public health experts, government policy makers, health workers, and information technologists.

For example, Afyadata was intended to be a surveillance technology for use among humans and animals to monitor common diseases in the 2 fields. Professionals from human and animal health, as well as software developers, extensively collaborated to elicit project needs and goals. Relevant government officials were also involved and endorsed the technology throughout all its development and implementation stages. As a result, Afyadata is now widely used for surveillance purposes in Tanzania and has been used in the surveillance of cholera cases, monitoring of hygiene and sanitation practices, early detection of Ebola in neighboring Democratic Republic of the Congo, and surveillance of diseases occurring among animals. Afyadata was also adopted for the screening of COVID-19 in neighboring Mozambique.

Similarly, Nigeria’s SORMAS followed a design thinking framework as a response to its precursor, the Sense Follow-up app. The Sense Follow-up app was an Android mobile technology rapidly developed in an emergency to support data collection during home visits following contacts of patients with Ebola for 21 days to document people who had been in contact with index patients with Ebola and support data collection during the first Ebola outbreak in Nigeria in 2014 [34,87]. However, the development was not sufficiently consultative because of time constraints imposed by the quick response time required to control Ebola. It was later deemed to have failed to meet the needs of the Ebola control team, specifically that it did not sufficiently support the bidirectional exchange of information; did not address case finding [57]; and had complex data manageability because of its modular architecture with separate systems for data storage, functions, and format and its interface [34,87]. The same team that developed the Sense Follow-up app then teamed up with philanthropists and public health and IT experts in designing SORMAS following a design thinking approach. The design thinking framework guided the SORMAS development team to identify system, user, and technical requirements and addressed all the shortcomings of the Sense Follow-up app. Although there is limited evidence of its evaluation, SORMAS is now a widely used technology for contact tracing various diseases in Nigeria. In addition, because it is easily customizable, SORMAS was central in response to COVID-19.

Similar to Afyadata and SORMAS, the technology for HIV case finding in the AMPATH program with clear objectives was able to screen thousands as had been originally planned [8]. It was designed to be reliable in resource-constrained settings, scalable to allow for screening of >2 million people in 3 years, and open-source software; easily integrate with other systems and devices; and have GPS capabilities. As a result, public health experts, software technologists, and government stakeholders contributed to successfully developing a technology to screen >1 million HIV contacts.

However, in Guinea, the Ebola contact tracing technology was developed using participatory processes and design thinking but lacked government policy maker buy-in, which led to suboptimal implementation, and most of the intended results were not achieved [32]. Tuberculosis HealthCheck and COVID-19 alert technology in South Africa did not have documented participatory development, but government support led to their nationwide rollout [46].

All technologies used in research studies were developed by research teams and did not have any documented use of design thinking frameworks or participatory development. These technologies were used to reach research objectives, and their tenure did not extend beyond the project. However, the research team in Uganda that implemented the trial by Davis et al [27] conducted a post hoc analysis of the trial using an implementation science framework, the Consolidated Framework for Implementation Research, to understand the reasons for the poor performance of the mHealth technology on their main outcome [59]. While not explicitly mentioning design thinking as a framework to follow, the researchers conceded that the lack of consultation with local stakeholders was a critical gap in development that may have deprived them of a robust outer setting, which is a critical indicator for successful implementation even in smaller research studies.

### Effectiveness of the mHealth Technologies and Reporting of Outcomes

Table 2 provides an overview of outcome reporting and technology development in the included studies. Technologies used in programmatic settings with a pretest-posttest setup were designed to reach specific targets and mainly reported outputs without comparisons, whereas research studies designed to answer a specific question reported both outputs and outcomes. Hence, effectiveness in the 2 approaches was defined differently. In technologies used for programmatic settings, effectiveness was defined as rolling out the technology to a large population and producing output. In research studies, effectiveness was defined as improving outcomes.

**Table 2:** Outcomes and app development.

Study title (design)	Output or outcomes	Development and evaluation
“Home-based tuberculosis contact investigation in Uganda: a household randomised trial” [27] (randomized study)	Primary outcome: completion of tuberculosis evaluation within 14 days of enrollment among contacts requiring additional evaluation for tuberculosis Secondary outcomes: Tuberculosis diagnosis and treatment initiation HIV diagnosis and linkage to HIV care	The app was developed by public health professionals with experience in implementing and evaluating mHealth^a^ technologies and by Dimagi CommCare specialists.
“Use of a mobile technology for Ebola contact tracing and monitoring in Northern Sierra Leone: a proof-of-concept study” [38] (cross-sectional study)	Number of Ebola contacts identified and traced per arm Time to find contact Ebola cases detected per arm Implementation feasibility	Developed by an information technologist in the United States working with the study team in Sierra Leone.
“Implementation of digital technology solutions for a lung health trial in rural Malawi” [6] (cross-sectional study)	Number of households located on the map	No app development; Google Earth was used to identify households.
“Using a mobile technology to Improve paediatric presumptive Tuberculosis identification in western Kenya” [41] (cross-sectional study)	Proportion of children recorded in the presumptive tuberculosis register Proportion of children testing positive for tuberculosis	The app was developed in Bangladesh and customized for use in this study.
“Evaluation of a Mobile Health Approach to Tuberculosis Contact Tracing in Botswana” [40] (pretest-posttest study)	Number of contacts screened using paper vs the app Time to complete screening using paper vs the app Missing and illogical data between paper and the app User satisfaction with paper vs the app	Researchers partnered with an IT firm to produce the contact tracing app.
“Feasibility of using a mobile app to monitor and report COVID-19-related symptoms and people’s movements in Uganda” [44] (pretest-posttest study)	Household members using the technology Household members reporting symptoms Household members referred for testing Household members diagnosed with COVID-19	Developed by a technology designer in consultation with officials from the Ministry of Health. No further details of the development process are given.
“The 117-call alert system in Sierra Leone: from rapid Ebola notification to routine death reporting” [36] (programmatic pretest-posttest)	Number of live Ebola alerts Number of death alerts Confirmed new cases from alerts	No app development, and a call center was repurposed to report suspected Ebola cases.
“A Smartphone App (AfyaData) for Innovative One Health Disease Surveillance from Community to National Levels in Africa: Intervention in Disease Surveillance” [47] (programmatic pretest-posttest)	Number of disease cases reported among humans and animals	Designed through a collaboration between public health and ICT^b^ specialists and government personnel. They developed a theory of change to guide development and implementation.
“Ebola virus disease contact tracing activities, lessons learned and best practices during the Duport Road outbreak in Monrovia, Liberia, November 2015” [35] (programmatic pretest-posttest)	The total number of contacts traced Total households found Total high- and low-risk contacts found Total symptomatic contacts Contact deaths recorded	No app development. The program used subpoenas to cell phone companies to provide location details of suspected Ebola contacts.
“Introduction of Mobile Health Tools to Support Ebola Surveillance and Contact Tracing in Guinea” [32] (programmatic pretest-posttest)	Total number of Ebola contacts monitored on CommCare IT and implementation challenges	An IT team developed it in the United States with local public health specialists in Guinea, local UN^c^ partners, and the government.
“USAID/South Africa Tuberculosis South Africa Project (TBSAP) Midterm Evaluation Report” [43] (programmatic pretest-posttest)	Not reported	Not reported
“Development and implementation of the Ebola Exposure Window Calculator: A tool for Ebola virus disease outbreak field investigations” [39] (programmatic pretest-posttest)	Exposure window for contact tracing to take place	Not reported
“Innovative Technological Approach to Ebola Virus Disease Outbreak Response in Nigeria Using the Open Data Kit and Form Hub Technology” [34] (programmatic pretest-posttest)	Number of users downloading the app Number of active users of the app	CDC^d^, WHO^e^, and DRC^f^ field teams and Johns Hopkins teams developed the technology, and the government endorsed its use.
“Evaluation of an Android-based mHealth system for population surveillance in developing countries” [8] (programmatic pretest-posttest)	Number of households visited for home-based counselling and testing and data collected using the app Time to complete screening using the app vs PDA App usability Cost of development and implementation	Members of the AMPATH^g^ project team developed the app in collaboration with staff from the Ministry of Health in Kenya. They had clear objectives for their scope and what they wanted the app to achieve.
“Evaluating the use of cell phone messaging for community Ebola syndromic surveillance in high-risk settings in Southern Sierra Leone” [33] (programmatic pretest-posttest)	Suspected Ebola cases Confirmed Ebola cases Reported deaths	No app development
“Implementing Surveillance and Outbreak Response Management and Analysis System (SORMAS) for Public Health in West Africa- Lessons Learnt and Future Direction” [37] (programmatic pretest-posttest)	Implementation outcomes from piloting the technology	Design thinking workshops were held in Nigeria and Germany to assess software requirements. Nigeria Centre for Disease Control and Prevention, Port Health, and other stakeholders were also involved in the design thinking methodology. Contact tracing requirements for other diseases, not just Ebola, were also considered in the design process.
“Using mHealth to self-screen and promote Tuberculosis awareness in Tanzania” [42] (programmatic pretest-posttest)	Number of people screened using the technology Number of presumptive cases screened using the app	Designed by the government and other implementors using the Ministry of Health mHealth platforms in a participatory process.
“TB HealthCheck puts tuberculosis self-screening in everyone’s hands ahead of World TB Day” [46] (programmatic pretest-posttest)	Not clearly stated	Not clearly stated
“Mobile health approaches to disease surveillance in Africa; Wellvis COVID triage tool” [45] (programmatic pretest-posttest)	Risk of COVID-19 after self-screenings	The development process is not described in detail.

^a^mHealth: mobile health.

^b^ICT: information and communications technology.

^c^UN: United Nations.

^d^CDC: Centers for Disease Control and Prevention.

^e^WHO: World Health Organization.

^f^DRC: Democratic Republic of the Congo.

^g^AMPATH: Academic Model Providing Access to Healthcare.

Of all the included studies, only the trial from Uganda randomly assigned participants to different groups, comparing outcomes objectively. The trial had primary outcomes of contacts completing tuberculosis evaluation within 14 days and secondary outcomes of treatment initiation and linking patients to care [27]. Contacts were assigned to the control, and SMS text messaging–facilitated interventions were at a household level. The trial showed that the mHealth technology had no effect on the primary outcome.

In addition, although they did not randomize participants or calculate sample size as in the randomized trial, the cross-sectional surveys and pretest-posttest studies included in this systematic review attempted to evaluate the performance of the mHealth technologies by reporting outcomes such as proportions of contacts diagnosed, and some compared performance between groups. For example, a study in Sierra Leone compared the number of Ebola contacts identified and traced between the Ebola contact tracing app and a paper-based data collection system using a conveniently selected sample [38]. The researchers also evaluated completion, the proportion of cases detected, and implementation feasibility between the 2 arms. Similarly, the study by Ha et al [40] also compared outcomes in proportions before and after implementation and in contrast to a paper-based system. This pretest-posttest study compared the number of contacts screened, time to complete an evaluation, and data quality between using a contact tracing app and paper-based systems [40]. Other cross-sectional studies only reported outcomes in one conveniently selected group [6,41], and one pretest-posttest study only reported outputs [44].

Programmatic setting studies predominantly reported immediate outputs or implementation experiences of the mHealth technologies. SORMAS, AMPATH, and Afyadata, which were the largest and most comprehensively developed technologies, only reported absolute numbers of the people screened and implementation experiences rather than actual outcomes to determine the performance of the mHealth technology. In addition, Tambua tuberculosis in Tanzania reported immediate outputs on tuberculosis screening but did not have an evaluation done on implementation outcomes. In South Africa, ConnecTB and tuberculosis HealthCheck, both used to facilitate tuberculosis screening, did not provide sufficient details on the outputs or evaluation of the technologies. Nevertheless, these technologies in programmatic settings reached large populations for contact tracing.

## Discussion

### Principal Findings

We reviewed the continuum of mHealth technologies used for contact tracing and case finding. We synthesized this information to improve understanding of mHealth’s value in contact tracing and inform how future projects can develop or implement it efficiently to improve contact tracing or implementation outcomes. Only 19 studies met the criteria and were included in the review. The technologies were developed by either customizing existing platforms or creating original software. Some technologies used to trace contacts did not require software development but used existing cellular network infrastructure. Technologies developed using design thinking frameworks with participatory activities had a higher likelihood of implementation fidelity. However, the effectiveness of these technologies was not sufficiently evaluated, and the outcomes of most technologies were only reported in small research studies.

Our search yielded a greater number of articles on studies using mHealth technologies for contact tracing than those obtained in previous reviews, likely reflecting the growing adoption of such technologies during the search period. In addition, our iterative search strategy, which featured relaxed criteria and encompassed a variety of infectious diseases, may have played a significant role in identifying this increased volume of articles. The review has also presented the entire pathway of using these technologies from the development stage until deployment and evaluation of outcomes. Our findings also confirm that mHealth outperforms traditional paper-based contact tracing regarding the timeliness of screening, data accuracy, and streamlining of the screening process. However, little evidence exists of mHealth’s impact or incremental value on contact tracing outcomes compared to paper because of underreporting. Despite this challenge, our study underscores the potential of using validated design frameworks during the developmental phase, which is likely to enhance the overall effectiveness of such solutions. Future public health projects that intend to develop and implement mHealth technologies can consider these insights as a guide into some of the prerequisites for implementing useful technologies that can meaningfully improve outcomes.

Our systematic review suggests that a more systematic approach to development using design thinking frameworks with participatory development, including buy-in from policy makers, could help technologies in achieving the intended case finding outputs and outcomes when compared to an approach that does not use these frameworks. For example, SORMAS was developed in Nigeria following design thinking and participatory processes involving public health experts, information technologists, and government officials. SORMAS was responding to an earlier mHealth app, Sense Follow-up, which had failed at the implementation stage due to not meeting user requirements because of poor development. Similarly, Afyadata in Tanzania and AMPATH in Kenya both involved extensive consultative processes, increasing their outputs. Contrastingly, even a collaboratively built technology may fail at implementation without government backing. For example, in Sierra Leone, Sacks et al [32] reported poor outputs from the Ebola contact tracing program, partly due to the lack of government officials’ commitment to supporting the program. In South Africa, ConnecTB did not document any design thinking frameworks or participatory development and was discontinued due to technical challenges despite having the policy makers’ support. None of the research studies documented the use of a design thinking framework, which may have affected their results, most of which were suboptimal, with the technology failing to show an impact on case finding. A post hoc analysis of the trial by Davis et al [27] also recommended using design thinking frameworks even in research studies to ensure that they are impactful [59].

In addition to using established frameworks in the development stage, financial resources were a significant factor in determining the extent of development and implementation. For example, among the technologies used in programmatic settings, SORMAS received an initial funding of €850,000 (US $929,509) from the European Union [85], Afyadata received US $450,000 from the Skoll Global Threats Fund [55], and the CommCare app for HIV screening was developed under a US $74.9–million US Agency for International Development–AMPATH program [86]. In addition, Tambua tuberculosis was government backed through Tanzania’s National Tuberculosis and Leprosy Programme [42]. Government involvement also assisted in obtaining buy-in from the ultimate end users of the technologies. However, compared to studies implementing technologies in programmatic settings, most research studies included in this review did not have the financial resources to execute the extensive development processes required for a technology to succeed. However, the dilemma is that well-funded programs tend to develop and implement large and sustainable technologies but often overlook comprehensive evaluations and, hence, fail to inform any future work meaningfully. Research studies, on the other hand, while rigorously evaluating their technologies, undergo a suboptimal development process due to limited funding. Consequently, despite restricted resources, research studies tend to use more robust evaluation methods in their assessments. For example, in Botswana, Ha et al [40] evaluated a pragmatic mHealth technology for tuberculosis contact tracing, but it was only implemented in a few settings, possibly due to limited funding, and the researchers could not conduct a large study to fully evaluate the program. Therefore, nesting evaluations in large programs as implementation research processes may be a way to improve evidence of mHealth technologies’ utility for contact tracing.

The primary focus of contact tracing programs is on improving case finding. However, there is also an expectation in some studies that improved case finding should also trickle down to the betterment of other outcomes down the tuberculosis care and treatment cascade, such as treatment initiation and completion [88,89]. However, using current evidence, it can be argued for mHealth technologies that, unless initially planned and incorporated in the design process, the desired contact tracing outcomes further down the cascade are inappropriate to determine the success of a contact tracing program and the effectiveness of mHealth technologies. Only 1 study in this review, a randomized trial, reported treatment initiation further down the contact tracing cascade [24,27], and the rest focused on outputs immediately after screening, such as numbers screened and testing positive. When using mHealth, immediate implementation outcomes such as technology development, implementation fidelity, feasibility, and acceptability could more appropriately measure effectiveness. This is because mHealth technologies can only measure what they are designed for. For instance, all technologies used in programmatic setting studies were designed to find and screen contacts but did not have modules developed for linkage to care, which would have allowed outcomes further down the cascade to be measured. Although this narrow focus on immediate outputs may limit the tenure of the technologies because they cannot demonstrate any value for the desired contact tracing outcomes, these outcomes are not determined only by finding the people with the disease. Instead, additional steps are required to optimize care linkage and treatment adherence. Small research studies with a limited scope cannot include all the steps from finding contacts, linking them to care, and showing a reduction in disease incidence, but without these steps, they may be too expensive and less likely to be adopted. Therefore, as discussed in the previous section, a participatory developmental process must be used to include such capabilities for mHealth and, where possible, develop the technology within a large well-funded program.

A cost analysis of the mHealth technology for contact tracing in Uganda found that this technology was underused because of its limited scope despite substantial investment in the development stages and initial implementation [90]. The authors suggested expanding the scope by increasing the volume of contacts served or expanding the use of the technology to the later stages of the contact tracing cascade. From this review, most technologies used to support Ebola contact tracing became defunct after the outbreak subsided, thus ultimately costing health programs because they were not used long enough to absorb or justify the costs of their development. Only SORMAS, which had an expanded scope beyond a single disease and activities beyond tracing, survived and continues to receive funding. Afyadata in Tanzania also has a large scope of contact tracing and surveillance activities in humans and animals and continues to be valuable to both health systems. Therefore, health programs should consider using technologies for contact tracing and case finding more broadly to capitalize on synergies and bring down costs to make them more sustainable. This will expand their scope and include multiple diseases or higher volumes of patients or expand the use of mHealth to all stages of the cascade, increasing the likelihood of the interventions’ sustainability. Investment cases for future mHealth technologies may be supported if there is evidence of their cost-effectiveness, and this may be necessitated by exploiting economies of scale and scope. Health economics studies and models have also shown that unit costs of interventions may decrease and be optimized for cost-effectiveness by implementing at the appropriate scale, within the right scope [91,92], and without compromising quality by exceeding these bounds [93].

### Limitations and Strengths of the Study

The primary limitation of our study stems from the context in which most of the reviewed mHealth technologies were deployed. Predominantly used within programmatic settings rather than through rigorous formal research, these technologies often lacked comprehensive outcome evaluations. Consequently, without these evaluations, our review faces challenges in definitively assessing the impact of mHealth technologies on enhancing contact tracing efforts. Despite this, the deployment of these technologies in well-funded programmatic settings offers valuable insights into the foundational requirements for successful contact tracing technologies, demonstrating how strategic funding can facilitate robust development and implementation.

In addition, our review faces limitations due to the unavailability of some key information, such as tenure of technology use beyond the initial studies, especially for technologies evaluated in research settings. The scarcity of detailed data in both primary literature and supplementary documents makes it difficult to assess whether the technologies were continued or discontinued after the research. This gap underscores the vital need for comprehensive documentation and rigorous reporting standards in the mHealth domain.

### Recommendations and Conclusions

The essential ingredients for developing functional and impactful mHealth technologies, whether for contact tracing or other health care interventions, include using a participatory design thinking framework, securing adequate funding, and establishing a clear plan for evaluating the implementation outcomes of the technology.

Many projects, especially research projects, often face challenges of limited funds to fully develop and implement their technologies. In such cases, an alternative approach can be integrating the development of mHealth technologies within larger technologies used in programmatic setting projects. This integration can provide the necessary resources and infrastructure to sufficiently develop and efficiently evaluate the technology. However, such ideal scenarios may not always be feasible, and the use of participatory design thinking frameworks becomes a prerequisite for developing an effective mHealth technology. This approach emphasizes collaboration with end users, stakeholders, and experts to ensure that the technology aligns with user needs, is inclusive, and considers the context-specific requirements.

## Appendix

Multimedia Appendix 1 Search terms.

Multimedia Appendix 2 Systematic review data extraction tool.

Multimedia Appendix 3 Quality assessment of the included studies.

Multimedia Appendix 4 Detailed explanation of each article in the systematic review.

Multimedia Appendix 5 PRISMA checklist.

## Abbreviations

AMPATH: Academic Model Providing Access to Healthcare
MeSH: Medical Subject Heading
mHealth: mobile health
ODK: Open Data Kit
PRISMA: Preferred Reporting Items for Systematic Reviews and Meta-Analyses
REDCap: Research Electronic Data Capture
SORMAS: Surveillance Outbreak Response Management and Analysis System
