# Supplementary material for: Using mHealth Technologies for Case Finding in Tuberculosis and Other Infectious Diseases in Africa: Systematic Review
Source: JMIR Mhealth Uhealth. 2024 Aug 26;12:e53211. doi: 10.2196/53211 (PMC11384173; doi:10.2196/53211)
Supplement: Multimedia Appendix 1 [file mhealth_v12i1e53211_app1.pdf]

Search terms

1 Pubmed

Term 1

(((mobile[All Fields] AND (("health"[MeSH Terms] OR "health"[All Fields]) OR ("technology"[MeSH Terms] OR "technology"[All Fields]) OR application[All Fields])) OR "ehealth"[All Fields] OR "telehealth"[All Fields] OR "telemedicine"[All Fields])) AND "tuberculosis"[MeSH Terms] AND (("contact"[All Fields] AND (tracing[All Fields] OR ("signs and symptoms"[MeSH Terms] OR ("signs"[All Fields] AND "symptoms"[All Fields]) OR "signs and symptoms"[All Fields] OR "finding"[All Fields]) OR investigation[All Fields])) OR (("CASE (Phila)"[Journal] OR "case"[All Fields]) AND (("signs and symptoms"[MeSH Terms] OR ("signs"[All Fields] AND "symptoms"[All Fields]) OR "signs and symptoms"[All Fields] OR "finding"[All Fields]) OR tracing[All Fields] OR investigation[All Fields]))) NOT ("systematic review"[pt])

Term 2

(("mobile health"[MeSH Terms] OR "mobile health"[All Fields] OR "mobile technology"[MeSH Terms] OR "mobile technology"[All Fields] OR "mobile application"[All Fields] OR "mobile application"[MeSH Terms] OR "ehealth"[All Fields] OR "ehealth"[MeSH Terms] OR "telehealth"[All Fields] OR "telehealth"[MeSH Terms] OR "telemedicine"[All Fields] OR "telemedicine"[MeSH Terms]) AND "tuberculosis"[MeSH Terms] AND ("contact tracing[All Fields] OR "contact tracing[MeSH Terms] OR "case finding"[All Fields] OR "case finding"[MeSH Terms] OR "contact investigation"[All Fields]OR "contact investigation"[MeSH Terms])) NOT (covid OR sars* OR ebola OR cancer)

Term 3

("tuberculosis" AND "contact tracing" AND "mHealth") NOT (covid OR ebola OR sars-cov*)

Term 4

(mobile OR digital) AND (health OR health technolog* OR intervention*) AND (tuberculosis) AND (contact AND (tracing OR investigation))

Term 5

(((("mobile" [All Fields] OR "digital" [All Fields]) AND (("health"[MeSH Terms] OR "health"[All Fields]) OR ("technology"[MeSH Terms] OR "technology"[All Fields]) OR application[All Fields])) OR "ehealth"[All Fields] OR "telehealth"[All Fields] OR "telemedicine"[All Fields])) AND "tuberculosis"[MeSH Terms] AND (("contact"[All Fields] AND (tracing[All Fields] OR ("signs and symptoms"[MeSH Terms] OR ("signs"[All Fields] AND "symptoms"[All Fields]) OR "signs and symptoms"[All Fields] OR "finding"[All Fields]) OR investigation[All Fields])) OR (("CASE (Phila)"[Journal] OR "case"[All Fields]) AND (("signs and symptoms"[MeSH Terms] OR ("signs"[All Fields] AND "symptoms"[All Fields]) OR "signs and symptoms"[All Fields] OR "finding"[All Fields]) OR tracing[All Fields] OR investigation[All Fields]))) NOT ("systematic review"[pt])

Term 6

(("tuberculosis"[MeSH Terms] OR "SARS-CoV-2"[MeSH Terms]) AND ("telemedicine"[All Fields] OR "mobile technology"[All Fields] OR "mHealth"[All Fields] OR "mobile health"[All Fields]) AND ("contact tracing"[MeSH Terms] OR ("contact tracing"[All Fields] AND "case finding"[All Fields]) OR "contact tracing"[All Fields]) AND ("2020/01/01"[PubDate] : "3000/12/31"[PubDate])) AND (("africa"[MeSH Terms] OR "africa"[All Fields]) OR ("africa south of the sahara"[MeSH Terms] OR ("africa"[All Fields] AND "south"[All Fields] AND "sahara"[All Fields]) OR "africa south of the sahara"[All Fields] OR ("sub"[All Fields] AND "saharan"[All Fields] AND "africa"[All Fields]) OR "sub-Saharan africa"[All Fields]))

Term 7

(((mobile[All Fields] AND (("health"[MeSH Terms] OR "health"[All Fields]) OR ("technology"[MeSH Terms] OR "technology"[All Fields]) OR application[All Fields])) OR "ehealth"[All Fields] OR "telehealth"[All Fields] OR "telemedicine"[All Fields])) AND ("tuberculosis"[MeSH Terms] OR "covid-19"[All Fields]) AND (("contact"[All Fields] AND (tracing[All Fields] OR ("signs and symptoms"[MeSH Terms] OR ("signs"[All Fields] AND "symptoms"[All Fields]) OR "signs and symptoms"[All Fields] OR "finding"[All Fields]) OR investigation[All Fields])) OR (("CASE (Phila)"[Journal] OR "case"[All Fields]) AND (("signs and symptoms"[MeSH Terms] OR ("signs"[All Fields] AND "symptoms"[All Fields]) OR "signs and symptoms"[All Fields] OR "finding"[All Fields]) OR tracing[All Fields] OR investigation[All Fields]))) NOT ("systematic review"[pt])

Term 8

(((((tuberculosis[Title] OR "tuberculosis"[MeSH Terms] OR "mycobacterium tuberculosis"[MeSH Terms] OR "tuberculosis, pulmonary"[MeSH Terms]) AND (("contact$"[All Fields]) OR ("contact tracing"[MeSH Terms]) OR "disease outbreaks"[MeSH Terms] OR "contact*"[Title] OR "spread"[Title] OR "contact screen*"[All Fields] OR "contact tracing"[Title] OR "disease transmission"[All Fields] OR "case find*"[Title] OR (cluster*[Title] AND analys*[Title]) OR "household*"[All Fields] OR "household contact*"[All Fields] OR ("case finding"[All Fields]) OR ("casefinding"[All Fields]) OR "case detection"[All Fields])))) AND ((("digital health"[All Fields] OR "digital technology"[All Fields]) OR ("telemedicine"[MeSH Terms] OR mHealth[Text Word])) OR ("cell phone"[MeSH Terms] OR "cell phone"[All Fields] OR "mobile phone"[All Fields] OR "mobile phone"[Text Word])))

PMC

Search term 1 (245)

Search: **("contact tracing" OR "active case finding" OR "case finding") AND ("mobile-health" OR mhealth OR "smartphone" OR cellphone) AND (HIV OR TB OR COVID OR Ebola OR infectious)** Sort by: **Most Recent**

("contact tracing"[All Fields] OR "active case finding"[All Fields] OR "case finding"[All Fields]) AND ("mobile-health"[All Fields] OR ("mhealth s"[All Fields] OR "telemedicine"[MeSH Terms] OR "telemedicine"[All Fields] OR "mhealth"[All Fields]) OR "smartphone"[All Fields] OR ("cell phone"[MeSH Terms] OR ("cell"[All Fields] AND "phone"[All Fields]) OR "cell phone"[All Fields] OR "cellphone"[All Fields] OR "cellphones"[All Fields])) AND ("hiv"[MeSH Terms] OR "hiv"[All Fields] OR "TB"[All Fields] OR ("sars cov 2"[MeSH Terms] OR "sars cov 2"[All Fields] OR "covid"[All Fields] OR "covid 19"[MeSH Terms] OR "covid 19"[All Fields]) OR ("hemorrhagic fever, ebola"[MeSH Terms] OR ("hemorrhagic"[All Fields] AND "fever"[All Fields] AND "ebola"[All Fields]) OR "ebola hemorrhagic fever"[All Fields] OR "ebola"[All Fields] OR "ebolavirus"[MeSH Terms] OR "ebolavirus"[All Fields]) OR ("infectious"[All Fields] OR "infectiousness"[All Fields]))

Translations

mhealth: "mhealth's"[All Fields] OR "telemedicine"[MeSH Terms] OR "telemedicine"[All Fields] OR "mhealth"[All Fields]

cellphone: "cell phone"[MeSH Terms] OR ("cell"[All Fields] AND "phone"[All Fields]) OR "cell phone"[All Fields] OR "cellphone"[All Fields] OR "cellphones"[All Fields]

HIV: "hiv"[MeSH Terms] OR "hiv"[All Fields]

COVID: "sars-cov-2"[MeSH Terms] OR "sars-cov-2"[All Fields] OR "covid"[All Fields] OR "covid-19"[MeSH Terms] OR "covid-19"[All Fields]

Ebola: "ebola's"[All Fields] OR "hemorrhagic fever, ebola"[MeSH Terms] OR ("hemorrhagic"[All Fields] AND "fever"[All Fields] AND "ebola"[All Fields]) OR "ebola hemorrhagic fever"[All Fields] OR "ebola"[All Fields] OR "ebolavirus"[MeSH Terms] OR "ebolavirus"[All Fields]

infectious: "infectious"[All Fields] OR "infectiousness"[All Fields]

Search term 2 (PubMed) (290)

Search: **("contact tracing" OR "active case finding" OR "case finding") AND ("mobile-health" OR mhealth OR "smartphone" OR cellphone)** Sort by: **Most Recent**

("contact tracing"[All Fields] OR "active case finding"[All Fields] OR "case finding"[All Fields]) AND ("mobile-health"[All Fields] OR ("mhealth s"[All Fields] OR "telemedicine"[MeSH Terms] OR "telemedicine"[All Fields] OR "mhealth"[All Fields]) OR "smartphone"[All Fields] OR ("cell phone"[MeSH Terms] OR ("cell"[All Fields] AND "phone"[All Fields]) OR "cell phone"[All Fields] OR "cellphone"[All Fields] OR "cellphones"[All Fields]))

Translations

mhealth: "mhealth's"[All Fields] OR "telemedicine"[MeSH Terms] OR "telemedicine"[All Fields] OR "mhealth"[All Fields]

cellphone: "cell phone"[MeSH Terms] OR ("cell"[All Fields] AND "phone"[All Fields]) OR "cell phone"[All Fields] OR "cellphone"[All Fields] OR "cellphones"[All Fields]

Search term 3 (PMC) (3368)

("contact tracing"[All Fields] OR "active case finding"[All Fields] OR "case finding"[All Fields]) AND ("mobile-health"[All Fields] OR ("telemedicine"[MeSH Terms] OR "telemedicine"[All Fields] OR "mhealth"[All Fields]) OR "smartphone"[All Fields] OR ("cell phone"[MeSH Terms] OR ("cell"[All Fields] AND "phone"[All Fields]) OR "cell phone"[All Fields] OR "cellphone"[All Fields])) AND (("hiv"[MeSH Terms] OR "hiv"[All Fields]) OR TB[All Fields] OR ("sars-cov-2"[MeSH Terms] OR "sars-cov-2"[All Fields] OR "covid"[All Fields] OR "covid-19"[MeSH Terms] OR "covid-19"[All Fields]) OR ("hemorrhagic fever, ebola"[MeSH Terms] OR ("hemorrhagic"[All Fields] AND "fever"[All Fields] AND "ebola"[All Fields]) OR "ebola hemorrhagic fever"[All Fields] OR "ebola"[All Fields] OR "ebolavirus"[MeSH Terms] OR "ebolavirus"[All Fields]) OR infectious[All Fields])

SCOPUS

ALL("contact tracing" OR "case finding") AND ALL(tuberculosis OR COVID OR HIV OR ebola OR "infectious disease") AND ALL("mobile health" OR "mhealth" OR "m-Health")

ALL("contact trac*" OR "case find*") AND ALL(tuberculosis OR COVID OR HIV OR ebola OR "infectious disease") AND ALL("mobile health" OR "mhealth" OR "m-Health" OR phone)

ALL(“contact trac*” OR “case find*”) AND ALL(tuberculosis OR COVID OR HIV OR ebola OR “infectious disease”) AND ALL(“mobile health” OR “mhealth” OR “m-Health” OR phone) AND (LIMIT-TO (PUBYEAR,2019) OR LIMIT-TO (PUBYEAR,2018) OR LIMIT-TO (PUBYEAR,2017) OR LIMIT-TO (PUBYEAR,2016) OR LIMIT-TO (PUBYEAR,2015) OR LIMIT-TO (PUBYEAR,2014) OR LIMIT-TO (PUBYEAR,2013))

Scopus search

ID	Name	Query	Documents	Date last run	Actions
#3	contact tracing case finding ...ulosis 896	ALL ("contact tracing" OR "case finding") AND ALL (tuber culosis OR covid OR hiv OR ebola OR "... View More	896	13 Dec 2021	+
#2	contact trac case find tuberculosis all	ALL ("contact trac*" OR "case find*") AND ALL (tuberculo sis OR covid OR hiv OR ebola OR "infe... View More	1,796	13 Dec 2021	+
#1	contact trac case find tuberculosis	ALL ("contact trac*" OR "case find*") AND ALL (tuberculo sis OR covid OR hiv OR ebola OR "infe... View More	328	13 Dec 2021	+

WEB OF SCIENCE

"contact tracing" OR "case finding" OR "screening" OR "infectious disease" OR "communicable disease" (All Fields) and mHealth OR "mobile Health" OR smartphone OR cell$phone OR "smart phone" OR "cell phone" (All Fields) and Article or Meeting Abstract or Early Access (Document Types) and Article or Meeting Abstract or Early Access or Proceeding Paper (Document Types) and SOUTH AFRICA or KENYA or GHANA or UGANDA or MADAGASCAR or TANZANIA or EGYPT or NIGERIA or DEM REP CONGO or ALGERIA or BOTSWANA or CAMEROON or ETHIOPIA or RWANDA or SIERRA LEONE or TUNISIA or LIBERIA or MALAWI or SENEGAL or SUDAN or ESWATINI or GUINEA or MOZAMBIQUE or REP CONGO or ZAMBIA or ZIMBABWE (Countries/Regions)

“contact tracing” OR “case finding” OR “screening” OR “infectious disease” OR “communicable disease” (All Fields) and mHealth OR “mobile Health” OR smartphone OR cell$phone OR “smart phone” OR “cell phone”

(All Fields) and Article or Meeting Abstract or Early Access (Document Types) and Article or Meeting Abstract or Early Access or Proceeding Paper (Document Types)

"contact tracing" OR "case finding" OR "screening" OR "infectious disease" OR "communicable disease" (All Fields) and mHealth OR "mobile Health" OR smartphone OR cell$phone OR "smart phone" OR "cell phone" (All Fields) and Article or Meeting Abstract or Early Access (Document Types) and Article or Meeting Abstract or Early Access or Proceeding Paper (Document Types) and SOUTH AFRICA or KENYA or UGANDA or MADAGASCAR or TANZANIA or EGYPT or NIGERIA or DEM REP CONGO or ALGERIA or BOTSWANA or CAMEROON or ETHIOPIA or RWANDA or SIERRA LEONE or TUNISIA or MALAWI or LIBERIA or SENEGAL or SUDAN or BURKINA FASO or ESWATINI or GUINEA or MOZAMBIQUE or NIGER or REP CONGO or ZAMBIA or ZIMBABWE (Countries/Regions) and SOUTH AFRICA or KENYA or UGANDA or MADAGASCAR or TANZANIA or EGYPT or NIGERIA or DEM REP CONGO or ALGERIA or BOTSWANA or ETHIOPIA or RWANDA or SIERRA LEONE or TUNISIA or LIBERIA or MALAWI or SENEGAL or BURKINA FASO or ESWATINI or GHANA or MOZAMBIQUE or NIGER or REP CONGO or ZAMBIA or ZIMBABWE (Countries/Regions)

“contact tracing” OR “case finding” OR “screening” OR “infectious disease” OR “communicable disease” (All Fields) and mHealth OR “mobile Health” OR smartphone OR cell$phone OR “smart phone” OR “cell phone” (All Fields)

"contact tracing" OR "case finding" OR "screening" (All Fields) and mHealth OR "mobile Health" OR smartphone OR cell$phone OR "smart phone" OR "cell phone" (All Fields)

"contact tracing" (All Fields) and mHealth OR "mobile Health" OR smartphone OR cell?phone (All Fields

"contact tracing" (All Fields) and mHealth OR "mobile?Health" OR smart?phone OR cell?phone (All Fields)

"contact tracing" (All Fields) and mHealth OR "mobile*Health" OR smart*phone OR cell*phone (All Fields)

2 Notes

i. The following article was dropped at the analysis stage Sense Follow-up: Using Technology to Organise Ebola Outbreak Data and Enable Effective Response by [59]. Sense follow-up was the same technology used in the following paper, “Innovative Technological Approach to Ebola Virus Disease Outbreak Response in Nigeria Using the Open Data Kit and Form Hub Technology” [41].

ii. The following article was dropped at the analysis stage because the (**Drop-out from the tuberculosis contact investigation cascade in a routine public health setting in urban Uganda: A prospective, multi-centre study)** because the CommCare technology was part of a larger trial **(Home-based tuberculosis contact investigation in Uganda: a household randomised trial)** which has also been discussed in this review.

iii. The Epi Info Viral Haemorrhagic Fever (VHF) Application: A Resource for Outbreak Data Management and Contact Tracing in the 2014-2016 West Africa Ebola Epidemic was also dropped at analysis because it was a database, not a contact tracing application. The eHealth Sense also used the VF Epi Info database to manage contact tracing data.
