# Supplementary material for: Using mHealth Technologies for Case Finding in Tuberculosis and Other Infectious Diseases in Africa: Systematic Review
Source: JMIR Mhealth Uhealth. 2024 Aug 26;12:e53211. doi: 10.2196/53211 (PMC11384173; doi:10.2196/53211)
Supplement: Multimedia Appendix 2 [file mhealth_v12i1e53211_app2.pdf]

Systematic review data extraction

The survey will take approximately 16 minutes to complete.

* Required

* This form will record your name, please fill your name.

1. Study title *

2. Year *

The value must be a number

3. First Author *

4. Meets inclusion criteria? *

- ◯ Yes
- ◯ No

5. Reason why criteria not met *

- ☐ No mHealth
- ☐ No contact tracing
- ☐ Not related to any disease in question
- ☐ Not in Africa
- ☐ Systematic review with irrelevant articles
- ☐ Parent study already included
- ☐ Systematic review include articles that have already been included
- ☐ Other

6. Disease type *

- [ ] Tuberculosis
- [ ] HIV
- [ ] COVID-19
- [ ] Ebola
- [ ] Other

7. Type of technology *

- [ ] Smartphone app
- [ ] USSD
- [ ] SMS
- [ ] Other

8. Study design *

- ◯ RCT
- ◯ Cohort
- ◯ Case control
- ◯ Implementation science/research
- ◯ Crossectional
- ◯ Programme
- ◯ Before and after
- ◯ Proposal
- ◯ Cluster randomised trial
- ◯ Proof-of-concept
- ◯ Systematic review
- ◯ Other

9. Study aim/s *

10. Study objectives *

11. Full text available *

- Yes
- No, abstract only
- Other

12. Duration of implementation period *

13. Is the a follow-up in the study? *

- Yes
- No

14. Duration of follow-up period *

15. Country *

16. Population *

17. Location of the study? *

- ◯ Facility-based
- ◯ Community-based
- ◯ Both community and facility
- ◯ Other

18. Name of the mHealth tech *

19. Description of mHealth application (The intervention) *

20. Type of phone or device compatible with mHealth tech *

21. Main purpose of the app? *

22. Screening modality *

- ◯ Symptom screening only
- ◯ Symptoms and specimen collection
- ◯ Symptom & referall for testing
- ◯ Unclear
- ◯ Other

23. Risk determination *

24. Comparison *

25. Outcomes *

26. Is application designed for contact tracing? *

- Yes
- No
- Other

27. Main target of the mHealth application *

- Health worker
- Patient/Individual
- Both patient & healtworker
- Other

28. Testing procedure *

- ◯ Rapid
- ◯ Sent to lab
- ◯ Clinical diagnosis
- ◯ No testing
- ◯ Not clearly stated
- ◯ Other

29. How the application was developed *

30. How participants were linked to health facilities *

31. Location of screening *

- [ ] Health facility
- [ ] Household
- [ ] Community
- [ ] Anywhere
- [ ] Other

32. How contact were identified *

33. Number of index patients found in intervention arm

The value must be a number

34. Number of index patients found in control arm

The value must be a number

35. Number of contacts listed in the intervention arm

The value must be a number

36. Number of contacts listed in the control arm

37. Number of contacts found from the listed in the intervention arm

The value must be a number

38. Number of contacts found from the listed in the control arm

The value must be a number

39. Number of contacts diagnosed with disease in the intervention arm

The value must be a number

40. Number of contacts diagnosed with disease in the control arm

The value must be a number

41. Number of diagnosed contacts linked to care in the intervention arm

42. Number of diagnosed contacts linked to care in the control arm

43. Describe the results

44. Reporting, monitoring and evaluation procedure.

45. Any challenges with the application and impact on results *

46. Notes

47. Final decision

- ◯ Keep
- ◯ Remove
- ◯ Unclear

48. Reason for exclusion

This content is neither created nor endorsed by Microsoft. The data you submit will be sent to the form owner.

Microsoft Forms
