# Supplementary material for: Using mHealth Technologies for Case Finding in Tuberculosis and Other Infectious Diseases in Africa: Systematic Review
Source: JMIR Mhealth Uhealth. 2024 Aug 26;12:e53211. doi: 10.2196/53211 (PMC11384173; doi:10.2196/53211)
Supplement: Multimedia Appendix 3 [file mhealth_v12i1e53211_app3.docx]

Supplementary material

Table 1: RoB 2 tool for assessing the included randomised trial (Response key: Y=Yes, N=No, PN=Probably Not, NA=Not Applicable, NI=Not Included)

| **Unique ID** | 17 | | **Study ID** | | | 17 | | | **Assessor** | DM |
| --- | --- | --- | --- | --- | --- | --- | --- | --- | --- | --- |
| **Ref or Label** | [1] | | **Aim** | | | adhering to intervention (the ‘per-protocol’ effect) | | | **The effect of adhering to intervention…** | failures in implementing the intervention that could have affected the outcome |
| **Experimental** | Intervention | | **Comparator** | | | Control | | | **Source** |  |
| **Outcome** | Completion of a full TB evaluation within 14 days | | **Results** | | | % Completing evaluation | | | **Weight** | 1 |
| ***Response key: Y=Yes, N=No, PN=Probably Not, NA=Not Applicable, NI=Not Included*** | | | | | | | | | | |
| **Domain** | | **Signalling question** | |  |  | | **Response** | **Comments** | | |
| **Bias arising from the randomisation process** | | 1.1 Was the allocation sequence random? | | | | | Y |  | | |
|  |  | 1.2 Was the allocation sequence concealed until participants were enrolled and assigned to interventions? | | | | | Y |  |  |  |
|  |  | 1.3 Did baseline differences between intervention groups suggest a problem with the randomisation process? | | | | | PN | Most of the household contacts` baseline characteristics were identical. However, the standard of care arm had a significantly higher proportion of contacts coughing for more than 2 weeks when compared to SOC (95% vs 70%, p=0.01) | | |
|  |  | **Risk of bias judgement** | | | | | **Some concerns** | Randomisation was done at the household level, and it was likely that the primary outcome would be altered by contamination. | | |
| **Bias due to deviations from intended interventions** | | 2.1 Were participants aware of their assigned intervention during the trial? | | | | | Y | Allocation was concealed up to the time of enrolment and was revealed after contact enrolment procedures had been completed. | | |
|  |  | 2.2 Were carers and people delivering the interventions aware of participants’ assigned intervention during the trial? | | | | | Y |  |  |  |
|  |  | 2.3. [If applicable:] If Y/PY/NI to 2.1 or 2.2: Were important non-protocol interventions balanced across intervention groups? | | | | | NA |  | | |
|  |  | 2.4. [If applicable:] Were there failures in implementing the intervention that could have affected the outcome? | | | | | Y | Complex logic required to generate SMSs, Software challenges in sending the SMSs, Low proportion submitting sputum, difficulty in providing sputum for home-based clients | | |
|  |  | 2.5. [If applicable:] Was there non-adherence to the assigned intervention regimen that could have affected participants’ outcomes? | | | | | NA |  | | |
|  |  | 2.6. If N/PN/NI to 2.3, or Y/PY/NI to 2.4 or 2.5: Was an appropriate analysis used to estimate the effect of adhering to the intervention? | | | | | Y |  | | |
|  |  | **Risk of bias judgement** | | | | | **Low** |  | | |
| **Bias due to missing outcome data** | | 3.1 Were data for this outcome available for all, or nearly all, participants randomised? | | | | | NI |  | | |
|  |  | 3.2 If N/PN/NI to 3.1: Is there evidence that result was not biased by missing outcome data? | | | | | N |  | | |
|  |  | 3.3 If N/PN to 3.2: Could missingness in the outcome depend on its true value? | | | | | NI |  | | |
|  |  | 3.4 If Y/PY/NI to 3.3: Is it likely that missingness in the outcome depended on its true value? | | | | | NI |  |  |  |
|  |  | **Risk of bias judgement** | | | | |  |  | | |
| **Bias in measurement of the outcome** | | 4.1 Was the method of measuring the outcome inappropriate? | | | | | Y |  | | |
|  |  | 4.2 Could measurement or ascertainment of the outcome have differed between intervention groups? | | | | | N |  | | |
|  |  | 4.3 Were outcome assessors aware of the intervention received by study participants? | | | | | Y |  | | |
|  |  | 4.4 If Y/PY/NI to 4.3: Could assessment of the outcome have been influenced by knowledge of intervention received? | | | | | Y | Randomisation was done at the household level, and it was likely that the primary outcome would be altered by contamination. Control group participants may have modified their behaviour to match those in the intervention because the latter received a better service. | | |
|  |  | 4.5 If Y/PY/NI to 4.4: Is it likely that assessment of the outcome was influenced by knowledge of intervention received? | | | | | Y |  |  |  |
|  |  | **Risk of bias judgement** | | | | | **Some Concerns** |  | | |
| **Bias in selection of the reported result** | | 5.1 Were the data that produced this result analysed in accordance with a pre-specified analysis plan that was finalised before unblinded outcome data were available for analysis? | | | | | Y |  | | |
|  |  | 5.2 ... multiple eligible outcome measurements (e.g., scales, definitions, time points) within the outcome domain? | | | | | NI |  | | |
|  |  | 5.3 ... multiple eligible analyses of the data? | | | | | NI |  | | |
|  |  | **Risk of bias judgement** | | | | | **Low** |  | | |
| **Overall bias** | | **Risk of bias judgement** | | | | | **Some concerns** | Randomisation at the household level may have contaminated the outcome. However, the use of the mHealth App may not have been affected. | | |

Table 2: Quality assessment using the Quality Assessment Tool for Before-After (Pre-Post) Studies with No Control Group

| **#** | **First author** | **Quality assessment questions** *(NA=Not Applicable, NR=Not Reported, CD=Can Not Determine)* | | | | | | | | | | | | |
| --- | --- | --- | --- | --- | --- | --- | --- | --- | --- | --- | --- | --- | --- | --- |
|  |  | **1** | **2** | **3** | **4** | **5** | **6** | **7** | **8** | **9** | **10** | **11** | **12** | **13** |
| **1** | [2] | Yes | NA | Yes | CD | CD | CD | Yes | NA | NA | Yes | No | NA | Good |
| **2** | [3] | Yes | No | Yes | No | Yes | Yes | Yes | No | NA | Yes | No | CD | Good |
| **3** | [4] | Yes | NA | Yes | CD | CD | Yes | Yes | NR | NA | Yes | NR | NR | Good |
| **4** | [5] | Yes | Yes | Yes | Yes | CD | Yes | Yes | No | NA | Yes | No | NA | Good |
| **5** | [6] | Yes | NR | Yes | CD | CD | Yes | No | No | NA | Yes | No | No | Fair |
| **6** | [7] | Yes | Yes | Yes | Yes | NR | Yes | CD | NA | NA | NA | NR | NA | Fair |
| **7** | [8] | Yes | NA | Yes | NA | CD | Yes | Yes | NA | NA | Yes | No | No | Fair |
| **8** | [9] | Yes | NA | NA | NA | NA | Yes | Yes | No | NA | Yes | No | No | Fair |
| **9** | [10] | Yes | Yes | Yes | Yes | NA | Yes | No | No | NA | Yes | No | No | Fair |
| **10** | [11] | Yes | No | Yes | CD | CD | CD | No | No | NA | Yes | Yes | No | Fair |
| **11** | [12] | Yes | NA | NR | NR | NR | Yes | CD | No | NA | CD | Yes | No | Fair |
| **14** | [13] | Yes | No | CD | NA | No | Yes | Yes | No | No | Yes | Yes | NA | Fair |
| **15** | [14] | Yes | NA | NA | NA | NA | CD | No | NA | NA | CD | CD | CD | Poor |
| **17** | [15] | Yes | NA | NR | NR | NR | NR | NR | NR | NA | NR | NR | NR | Poor |
| **18** | [16] | Yes | NA | CD | NA | NA | Yes | Yes | NA | NA | NA | No | No | Poor |
| **Key to quality assessment questions** | | | | | | | | | | | | | | |
| **1** | Was the study question or objective clearly stated? | | | | | | | | | | | | | |
| **2** | Were eligibility/selection criteria for the study population pre-specified and clearly described? | | | | | | | | | | | | | |
| **3** | Were the participants in the study representative of those who would be eligible for the test/service/intervention in the general or clinical population of interest? | | | | | | | | | | | | | |
| **4** | Were all eligible participants that met the pre-specified entry criteria enrolled? | | | | | | | | | | | | | |
| **5** | Was the sample size sufficiently large to provide confidence in the findings? | | | | | | | | | | | | | |
| **6** | Was the test/service/intervention clearly described and delivered consistently across the study population? | | | | | | | | | | | | | |
| **7** | Were the outcome measures pre-specified, clearly defined, valid, reliable, and assessed consistently across all study participants? | | | | | | | | | | | | | |
| **8** | Were the people assessing the outcomes blinded to the participants’ exposures/interventions? | | | | | | | | | | | | | |
| **9** | Was the loss to follow-up after baseline 20% or less? Were those lost to follow-up accounted for in the analysis? | | | | | | | | | | | | | |
| **10** | Did the statistical methods examine changes in outcome measures from before to after the intervention? Were statistical tests done that provided p values for the pre-to-post changes? | | | | | | | | | | | | | |
| **11** | Were outcome measures of interest taken multiple times before the intervention and multiple times after the intervention (i.e., did they use an interrupted time-series design)? | | | | | | | | | | | | | |
| **12** | If the intervention was conducted at a group level (e.g., a whole hospital, a community, etc.), did the statistical analysis take into account the use of individual-level data to determine effects ... | | | | | | | | | | | | | |
| **13** | Quality Rating | | | | | | | | | | | | | |

Table 3: Quality assessment of cross-sectional studies (NA=Not Applicable, NR=Not Reported, CD=Can Not Determine)

| Questions | [17] | [18] | [19] |
| --- | --- | --- | --- |
| Was the research question or objective in this paper clearly stated? | Yes | Yes | Yes |
| Was the study population clearly specified and defined? | Yes | Yes | Yes |
| Was the participation rate of eligible persons at least 50%? | NA | Yes | CD |
| Were all the subjects selected or recruited from the same or similar populations (including the same time period)? Were inclusion and exclusion criteria for being in the study pre-specified and App... | Yes | Yes | Yes |
| Was a sample size justification, power description, or variance and effect estimates provided? | No | CD | CD |
| For the analyses in this paper, were the exposure(s) of interest measured prior to the outcome(s) being measured? | NR | Yes | Yes |
| Was the timeframe sufficient so that one could reasonably expect to see an association between exposure and outcome if it existed? | NR | Yes | Yes |
| For exposures that can vary in amount or level, did the study examine different levels of the exposure as related to the outcome (e.g., categories of exposure, or exposure measured as continuous | NR | NR | CD |
| Were the exposure measures (independent variables) clearly defined, valid, reliable, and implemented consistently across all study participants? | NA | Yes | Yes |
| Was the exposure(s) assessed more than once over time? | NA | No | No |
| Were the outcome measures (dependent variables) clearly defined, valid, reliable, and implemented consistently across all study participants? | NA | Yes | Yes |
| Were the outcome assessors blinded to the exposure status of participants? | NA | No | No |
| Was loss to follow-up after baseline 20% or less? | NA | NA | NA |
| Were key potential confounding variables measured and adjusted statistically for their impact on the relationship between exposure(s) and outcome(s)? | NA | NR | CD |
| Quality Rating | Fair | Good | Good |
| Any comments? | They did not report any outcomes from the home visits but extensively discussed how the applications were used to find and trace homes. | While the paper can be termed good, the authors somewhat reported the application results better than paper-based. |  |

1. Davis JL, Turimumahoro P, Meyer AJ, Ayakaka I, Ochom E, Ggita J, et al. Home-based tuberculosis contact investigation in uganda: A household randomised trial. ERJ Open Research. 2019;5(3). PMID:31367636. doi:10.1183/23120541.00112-2019.

2. Jia K, Mohamed K. Evaluating the use of cell phone messaging for community ebola syndromic surveillance in high risked settings in southern sierra leone. African Health Sciences. 2015;15(3):797-802. PMID:26957967. doi:10.4314/ahs.v15i3.13.

3. Rajput ZA, Mbugua S, Amadi D, Chepngeno V, Saleem JJ, Anokwa Y, et al. Evaluation of an android-based mhealth system for population surveillance in developing countries. Journal of the American Medical Informatics Association : JAMIA. 2012;19(4):655-9. PMID:22366295. doi:10.1136/amiajnl-2011-000476.

4. Diaz N, Moturi E. Using mhealth to self-screen and promote tb awareness in tanzania. Tanzania: Challenge TB; 2019.

5. Ha YP, Tesfalul MA, Littman-Quinn R, Antwi C, Green RS, Mapila TO, et al. Evaluation of a mobile health approach to tuberculosis contact tracing in botswana. J Health Commun. 2016;21(10):1115-21. PMID:27668973. doi:10.1080/10810730.2016.1222035.

6. Karimuribo ED, Mutagahywa E, Sindato C, Mboera L, Mwabukusi M, Kariuki Njenga M, et al. A smartphone app (afyadata) for innovative one health disease surveillance from community to national levels in africa: Intervention in disease surveillance. JMIR Public Health and Surveillance. 2017;3(4):e94. PMID:29254916. doi:10.2196/publichealth.7373.

7. Adeoye OO, Tom-Aba D, Ameh CA, Ojo OE, Ilori EA, Gidado SO, et al. Implementing surveillance and outbreak response management and analysis system (sormas) for public health in west africa-lessons learnt and future direction. International Journal of Tropical Disease & Health. 2017;22(2):1-17. doi:10.9734/IJTDH/2017/31584.

8. Alpren C, Jalloh MF, Kaiser R, Diop M, Kargbo S, Castle E, et al. The 117 call alert system in sierra leone: From rapid ebola notification to routine death reporting. BMJ Global Health. 2017;2(3):e000392. PMID:28948044. doi:10.1136/bmjgh-2017-000392.

9. Whitesell A, Bustamante ND, Stewart M, Freeman J, Dismer AM, Alarcon W, et al. Development and implementation of the ebola exposure window calculator: A tool for ebola virus disease outbreak field investigations. PLoS One. 2021;16(8):e0255631. PMID:34352008. doi:10.1371/journal.pone.0255631.

10. Wolfe CM, Hamblion EL, Schulte J, Williams P, Koryon A, Enders J, et al. Ebola virus disease contact tracing activities, lessons learned and best practices during the duport road outbreak in monrovia, liberia, november 2015. PLoS Neglected Tropical Diseases. 2017;11(6):e0005597. PMID:28575034. doi:10.1371/journal.pntd.0005597.

11. Tom-Aba D, Olaleye A, Olayinka AT, Nguku P, Waziri N, Adewuyi P, et al. Innovative technological approach to ebola virus disease outbreak response in nigeria using the open data kit and form hub technology. PLoS One. 2015;10(6):e0131000. PMID:26115402. doi:10.1371/journal.pone.0131000.

12. Sacks JA, Zehe E, Redick C, Bah A, Cowger K, Camara M, et al. Introduction of mobile health tools to support ebola surveillance and contact tracing in guinea. Glob Health Sci Pract. 2015;3(4):646-59. PMID:26681710. doi:10.9745/GHSP-D-15-00207.

13. Mugenyi L, Nsubuga RN, Wanyana I, Muttamba W, Tumwesigye NM, Nsubuga SH. Feasibility of using a mobile app to monitor and report covid-19 related symptoms and people's movements in uganda. PLoS One. 2021;16(11):e0260269. PMID:34797878. doi:10.1371/journal.pone.0260269.

14. URC. Usaid/south africa tuberculosis south africa project (tbsap) midterm evaluation report. 2020.

15. Praekelt.org. Tb healthcheck puts tuberculosis self-screening in everyone’s hands ahead of world tb day: Praekelt.org; 2021 [cited 2023 May 8, 2023]. Available from: <https://www.praekelt.org/news>.

16. Owoyemi A, Ikpe R, Toye M, Rewane A, Abdullateef M, Obaseki E, et al. Mobile health approaches to disease surveillance in africa; wellvis covid triage tool. Digital Health. 2021;7:2055207621996876. PMID:33680485. doi:10.1177/2055207621996876.

17. Chisunkha B, Banda H, Thomson R, Squire SB, Mortimer K. Implementation of digital technology solutions for a lung health trial in rural malawi. Eur Respir J. 2016;47(6):1876-9. PMID:27076597. doi:10.1183/13993003.00045-2016.

18. Danquah LO, Hasham N, MacFarlane M, Conteh FE, Momoh F, Tedesco AA, et al. Use of a mobile application for ebola contact tracing and monitoring in northern sierra leone: A proof-of-concept study. BMC Infect Dis. 2019;19(1):810. PMID:31533659. doi:10.1186/s12879-019-4354-z.

19. Szkwarko D, Amisi JA, Peterson D, Burudi S, Angala P, Carter EJ. Using a mobile application to improve pediatric presumptive tb identification in western kenya. Int J Tuberc Lung Dis. 2021;25(6):468-74. PMID:34049609. doi:10.5588/ijtld.20.0890.
