# Supplementary material for: Using mHealth Technologies for Case Finding in Tuberculosis and Other Infectious Diseases in Africa: Systematic Review
Source: JMIR Mhealth Uhealth. 2024 Aug 26;12:e53211. doi: 10.2196/53211 (PMC11384173; doi:10.2196/53211)
Supplement: Multimedia Appendix 4 [file mhealth_v12i1e53211_app4.docx]

# Summary of the applications

In the appendix, we summarise the articles retained after the full-text review[1-19].

## In-house software platforms

### Surveillance, Outbreak Response Management and Analysis System (SORMAS) [6]

SORMAS was developed in 2015 Nigeria’s Centre for Disease Control (NCDC) with EUR 850 000 funding from the European Union through Helmholtz Centre for Infection Research in Germany [20]. It was mainly conceived as a surveillance tool for a group of infectious diseases among humans and animals and as a response to limitations identified in ODK [6]. The public health staff from the NCDC collaborated with developers from Germany and the United States of America to create a technology that, unlike ODK, could simultaneously conduct surveillance among humans and animals, allowed easy data management by not allowing easy two-way data flow between field workers and real-time monitoring and evaluation. This grouping of experts was a new approach as opposed to the when they developed Sense Follow. SORMAS has now been implemented in Ebola surveillance in Nigeria and expanded to Ghana with new modules addressing other diseases such as TB. In its timeline, the Nigerian government has adopted SORMAS as the official surveillance tool, covering a population of more than 200 million people during the COVID-19 pandemic in 2020. It has also been previously used in outbreaks of monkeypox, bacterial meningitis and Lassa fever in Nigeria [21]. Design thinking was critical to the application’s success, which captures and addresses the context in which the application will be used and ensures that it is fit for its purpose [20, 22]. However, the main limitation with SORMAS is the failure to conduct and publish an evaluation despite the successes. Currently, available literature focuses on its acceptability, usability, and aspects which it does better than other software. However, more evidence is required on how its use has impacted actual outcomes on case finding.

### Ebola Exposure Window Calculator[8]

Effective Ebola control partly requires knowledge of the source of infections and transmission chains. However, outreach workers often face challenges when using paper-based systems to estimate the windows, and they may fail to establish sources of infection and the transmission chains. The Ebola Exposure Window Calculator was developed in the Democratic Republic of Congo by the Centers for Disease Control (CDC) with the primary objective of replacing a paper-based system that was sub-optimal in calculating exposure windows for Ebola contacts. The application was developed for Android and Apple operating systems and deployed as open-source software on both platforms. Although aimed at outreach workers, any user could download and use the application on both platforms and use it at no cost, provided they had data bundles. After endorsement by the ministry of health, the Ebola Virus Diseases (EVD) outreach workers were trained on using the App. They started calculations using the application to estimate exposure windows during contact tracing to determine the risk of infection. The application was designed to also capture details such as the contacts` symptoms and deaths during the household visits. The algorithms built into the application ensured that tracers made consistent decisions and only needed an estimated date of when the symptoms began for the estimate to be produced. A significant limitation of the application was that it required developing a secondary application to establish the transmission chains. Also, the application was not evaluated against any health outcomes except its use in different countries. The exposure window calculator as a standalone application is also unlikely to be relevant in less acute diseases, and thus its sustainability is questionable [23].

### Presumptive paediatric TB mobile Android application (PPTBMAPP) [12]

PPTBMAPP was initially developed in Pakistan under Wave 6 TB reach project and used by CHW in waiting areas of health facilities [12]. The App was aimed at improving the identification of presumptive TB among children. The App was then adopted in Kenya in 2018. It was implemented with a USD26,000 grant for the same purpose as in Pakistan and evaluated its usability and quantitative outcomes. Details of the development have not been published, but results from implementation in Kenya showed a significant increase in paediatric presumptive cases within the 6-month piloting period from August 2019 to January 2020. The presumptive TB cases which were only 10.7% before the intervention and increased to 16.2% after using the App (p-value<0.001). However, no significant increase in active TB was identified despite a 3.6% absolute increase pre-implementation. The use of the application was found feasible and acceptable by the CHWs. However, there were several limitations, such as the length of the study, small sample size and adaptation of the App to local needs, which may have influenced the desired outcome of significantly improving case identification. The authors believe a more extensive study comparing the mobile application with a paper-based system will be worthwhile.

### ConnecTB [14]

The ConnecTB application was developed for the TB South Africa programme (TBSAP), which was implemented by the University Research Council (URC) and funded by USAID. In 2015, URC developed ConnecTB to facilitate the recording and reporting of patient data by outreach workers during their support visits to TB patients. It was first piloted in the Eastern Cape province of South Africa, where outreach teams would then take advantage of their presence in the homes to conduct TB screening of household contacts. The development process of the ConnecTB application is not available in the public domain. There is also no formal evaluation of the ConnecTB application on contact tracing outcomes. While every URC report has contact tracing outputs for the indicator to increase contact tracing of key populations, none of them has explicitly evaluated the application. However, an April 2020 quarterly project report for the overall TBSAP project showed that further application development had been halted. Another mid-term report in the same year classified it as a less effective intervention [14]. Although ConnecTB, in its pilot phases, had shown positive outcomes in improving mainly on MDR-TB adherence, it faced technical challenges during scale up to other districts leading to its halting. As a result, a search for a new provider and platform was initiated. The TBSAP has since closed, and ConnecTB is no longer used in the country.

### Wellvis [16]

The Wellvis app was a rapidly developed app (over two weeks) in Nigeria which was used for symptom screening for COVID-19. It was a “public-facing” app which allowed users to report their symptoms over a web application or through an Unstructured Supplementary Service Data (USSD). At the end of the screening process, the App would give a risk rating without mentioning the subsequent steps. Also, the results of the screening outputs are not presented.

## Existing applications; ODK, CommCare, Kobo and Epi Info

ODK and CommCare were the most popular platforms in 8 of the 19 studies, and one used Kobo Toolkit [15].

### ODK

ODK was used in the following projects on Ebola [3], HIV [17], multiple diseases [19] and two TB [9, 10]. The oldest was the HIV project, where the application was deployed to support HIV screening in 2010.

#### Sense Follow-up [3]

Sense follow-up was implemented during Nigeria’s 2014 Ebola outbreak to support data collection during screening symptoms for Ebola. Sense follow-up was a pre-cursor to SORMAS [22], the latter eventually becoming the country’s mainstream mHealth tool for Ebola. The application was developed to overcome challenges with paper-based data collection such as large volume of data capturing, delays in relaying of information, poor turnaround time of lab results, and physical constraints with vehicle traffic in the city when consolidating paper-based data collection forms and slow communication between the teams. The development team (which also later developed SORMAS) used ODK collect and Form Hub technologies with other supporting technologies, namely, a dashboard for reporting, monitoring and evaluation, and ArcGIS for location households. Contact tracers used smartphones to screen for Ebola symptoms, while laboratory and case management staff used tablets to view and monitor data as it was being transmitted from the field. Sense follow-up functioned in real-time such that case managers and other senior staff would be immediately alerted, via text messaging, of an adverse finding like a contact’s temperature above 37°C and promptly activate an evacuation plan. A similar interaction would also occur for the laboratory requisitions and later used for real-time reporting of results. A separate application, eHealth sense, was also developed to follow up and monitor contact for 21 days.

The challenges identified included the costs of setting up the technology and procuring the devices, the need for high-speed internet to allow data transmission, and integrating the Form Hub structure with other applications. The application has since been discontinued, and the link on the Google Play Store also shows that it is no longer available for download [Sense Follow-up Android App](https://play.google.com/store/apps/details?id%92=%92com.ehealthafrica.senseebola).

These technologies improved daily reporting of field activities to 100%, reduced turnaround times for evacuating symptomatic contact to 1 hour from between 3 to 6 hours and improved turnaround times for laboratory results. In addition, contact evacuations broke the transmission chain, which is acute in Ebola. Data accountability improved because of GPS tracking and tagging of the data collectors. Incident managers could also make prompt informed decisions due to the availability of data and summaries on the dashboard.

#### Household mapping in Malawi [9]

In Malawi, ODK was used with Google Earth Pro to locate and enumerate households in preparation for a chronic airways disease and tuberculosis trial [9, 24]. On Google Earth Pro, catchment areas were demarcated, clusters drawn, and potential households identified. These activities were done in a desktop review without needing to visit the homes. The coordinates of the identified households were uploaded into smartphones which research assistants used to locate households and calculate the travelling distances. This mapping technology successfully traced households without prior contact with its members. In the home, research assistants used ODK as a data collection tool to capture responses from the interviewers. However, it is unclear what interviews were conducted in the households or if they related to contact tracing. Also, information on the development of the ODK application is not detailed. The study, however, gives perspective to the usefulness of mapping technologies to located households. Two main challenges identified in the exclusive use of mapping were research assistants visiting inhabited structures (like churches and farm barns) that the analysis on the map had identified and how to reach remote and difficult-to-access areas which had appeared accessible on the map. The challenges were overcome by recalibrating the map and enlisting help from local guides, which is likely a crucial factor to consider when tracing virtually mapped households.

#### Botswana TB contact tracing app [10]

In 2013, Ha, Tesfalul [10] conducted a small pilot study in Botswana to compare a new electronic contact tracing application developed on ODK to the traditional paper-based system. The researchers first collected data from March to September 2012 using a paper-based contact tracing system at selected facilities in Gaborone and Kweneng districts for six months. The paper-based system had been in place since 2009. After the paper-based phase, they introduced a mHealth application for another six months, from September 2012 to March 2013, using the same team of data collectors. The mobile application used the same questions from the screening tool for adults and paediatrics. The tool captured start and end times, built-in checks for errors, missing data, illogical responses, and captured geographic coordinates. Data collection did not require an internet connection, but synchronisation was done when an internet connection was restored. This meant that screening could still be conducted in poor network areas.

The mobile contact tracing application screened 137 more contacts than the paper-based system. Data for 12 contacts from the paper-based system could not be evaluated for screening duration due to missing start and end times, whereas the mobile application data were all complete. The time required for an adult screening session was half the time needed using paper. About 10% of adult contacts` and up to 80% of paediatric contacts` data had more than one missing or illogical value, while none were observed on the application. The end users rated the application better on system usefulness, quality of information and interface. However, no other data is available on the actual TB diagnosed when screening was conducted using paper compared to the application. No evidence exists of adopting this application by the national TB programme in Botswana. There have, however, been reviews and proposals for similar technologies whose implementation has not materialised or is not documented [25-27].

#### USAID Academic Model Providing Access to Healthcare (AMPATH) HIV screening programme[17]

In 2010 in Kenya, the USAID under the AMPATH programme developed, deployed and evaluated an Android application *with a 3-year target of visiting and screening 2 million individuals for HIV through home-based counselling and testing (HCT), collecting basic health information, offering rapid HIV testing, collecting sputum from individuals at risk of tuberculosis, and offering other services if needed in the household.* [17]. The PPTBMAPP application discussed under the in-house software platforms was also successfully implemented in the later phases of the AMPATH programme in Kenya [12]. The AMPATH programme used personal digital assistance (PDA) devices as far back as 2009 in task shifting for decanted HIV patients where community caregivers would use pre-programme PDAs for follow-ups [28, 29]. The PDAs had already been evaluated and found that they outperformed paper [30] but could not be used on a large scale and on multiple devices, which was needed for the community-based screening. Other challenges with the PDA included their costs, data integration challenges with electronic medical records, which require additional work, poor connection on PDAs, especially for GPS marking, and limited functionality of the devices for more sophisticated functions such as scanning of barcodes [17].

ODK collect ticked all the boxes necessary to support the screening of over 2 million households because it could be implemented on a large scale, supported multiple questions and data types, had GPS functionality, could capture and store media, and scan barcodes. The new Android application was developed, and usability testing was conducted among end-users. Within six months of the initial piloting phase with 70 outreach workers, 18850 had been visited, and testing completed for 63,470 people. Comparing the Android application to the PDAs, 85% found the former to be faster, 89% said it was easier to use, and 84% confirmed that the Android application resulted in better data quality. About 80% felt that it facilitated their interactions better than PDAs, while 89% wished to continue using the Android application. Although the Android application was successful, the programme implementers found that only 28% of the people testing positive for HIV presented for further care at the clinics. Therefore, finding the people with the App alone is insufficient to link them to care, and other strategies down the cascade were proposed, such as text message reminders. A key factor to the success of this programme was the commitment from the Ministry of Health in Kenya.

#### AfyaData [19]

AfyaData was developed in Tanzania by a multidisciplinary team of animal and human health experts as a surveillance tool. The development of AfyaData was motivated by inefficiencies with paper-based systems as described in other technologies. However, it was further discovered that about 70% of human diseases had origins in animals, and thus, a solution to prevent diseases among humans should also include animal surveillance. The initial concept also proposed a contact tracing module but not built into the application. The application was developed by The Southern African Centre for Infectious Disease Surveillance (SACIDS) through an EpiHack, a collaborative process between information technologists and health experts to create scalable and sustainable digital solutions for disease prevention [19, 31]. The development also followed design thinking theory which, like in SORMAS development [22], prioritised the end-user experience. Implementing AfyaData was guided by a theory of change (TOC) conceptualised during the participatory EpiHacks. The components of the TOC included a participatory model which housed an innovation hub among the health and IT experts to continue discussing ways to improve the application, improvement and growth of technical capabilities of the users of the application and a long-term view of what the application must focus on to ensure its sustainability within the country and across the borders of Tanzania [19].

AfyaData worked like other ODK-based applications described in the previous sections. However, a smartphone application would be used instead of paper to capture data and immediately relayed to senior personnel for monitoring and evaluation. Unique to AfyaData was that it did not require an internet connection for collecting data because data could be synchronised when a connection was established. Another unique feature was the integrated human and animal modules within a single application. The Tanzanian government also used the application to control a cholera outbreak in the country [32]. Like Ebola, Cholera requires quick turnaround times through screening and identifying people at risk. By 2017, more work was being planned using customised versions of AfyaData in Kenya, Uganda, Malawi, and Botswana [32]. In 2021, AfyaData was also featured in Gates Notes as one of the heroic applications for responding to outbreaks [33].

### CommCare

CommCare is a paid application developed by Dimagi (CommCare, Dimagi, Cambridge, MA, USA). The CommCare website describes it as an ICT tool for development, utilised by monitoring, evaluation, and research teams for data collection and tracking, suitable for both small and large-scale office operations. CommCare also enables the logical customising of digital workflows for field workers for intuitive data collection. CommCare uses ODK collect as its base software [34]. In addition, three included studies use CommCare as a data collection and contact tracing tool.

#### Ebola contact tracing application and Tableau dashboard in Guinea [1],

The National Ebola Coordination Unit in Conakry, Guinea responded to the Ebola outbreak in 2014 with a solution that would improve quick identification of Ebola, promptly evacuate them, and follow up within 21 days. As observed in other studies, the use of paper-based systems was suboptimal and did not meet the urgency required to control Ebola. They developed a contact tracing application on CommCare because the system allowed longitudinal tracking over time, reassigned contacts between tracers and had a history of use in the country’s maternal and child health programmes [1]. The application followed the contact tracing steps, which began with registering contacts in the household, following contacts for 21 days and closing the contact system after the 21 days based on an outcome. The programme had three major steps, which were completed within 8 weeks; preparation for 5 weeks where the application and dashboards were conceptualised and developed, deployment for 3 weeks for procurement, training and the first use of the application, and then adaption for an initial. As of May 2015, over 9000 contacts were monitored on the CommCare application from five territories. Conakry had the highest number of contacts with 6,151 over 5.5 months, the longest deployment period. The Tableau dashboard, which Dimagi recommended for its ease of integration, helped with real-time monitoring and evaluation and quick response events.

However, challenges were identified at various stages of the implementation, some of which were the architecture of the technology. First, the evaluation found that CommCare was not suited for responding to emergencies such as Ebola, whose progression is dynamic and requires continuous backend editing. Such is a feature of most applications built on existing software because end users are limited in what they can edit. Therefore, the Nigerian team built SORMAS and stopped using ODK, on which CommCare is also based. Second, although Dimagi recommended using Tableau, there was poor implementation fidelity because the data structure from CommCare was not always compatible with Tableau. There were also challenges with using smartphones by older contact tracers unfamiliar with technology, which led to data errors and, in some instances, reverted to paper-based systems. Some community members also did not trust the data collected on the application, and the contact tracer would revert to paper. Offline data collection did not help in some very remote areas, and data had to be submitted after several days when tracers moved to areas with a network. Another major challenge in the CommCare programme was that contact tracers were co-managed by different partners and organisations, resulting in poor leadership clarity despite an attempt to create consistent policies. There was also an insufficient political commitment from the government as to where officials had to complete interests which hindered them from efficiently using the data.

Consequently, because of these, the use of CommCare in an emergency in Guinea did not work as expected. The mobile application did not replace paper as expected. There were still questions from the multiple organisations involved on the value of introducing such technologies to a population with low literacy levels.

#### Ebola Contact Tracing application (ECT app) in Sierra Leone [7],

Implementing the ECT app highlights the importance of developing sustainable applications that can be used in multiple diseases. Like in Guinea, CommCare was also used to develop a contact tracing application in Sierra Leone but is now evaluated in a randomised controlled trial. In this study, a cluster randomised trial was planned to evaluate the effectiveness of mHealth applications compared with paper-based systems for Ebola contact tracing [7]. However, the number of Ebola cases rapidly declined, and the study only became a proof of concept, which made the application obsolete after the evaluation. For the results, however, the paper-based option found 408 contacts from 25 patients (16.32 contacts per patient), and the ECT app found 646 contacts from 18 patients (35.9 contacts per patient). The ECT app outperformed the paper-based system in terms of completion of screening and data quality. However, only 36% of the daily reporting forms in the paper system were returned, and less than half of the returned contacts were monitored for the full 21 days. The authors identified their major challenge as developing an application that suits the need to respond to an emergency.

#### Uganda TB contact tracing [11],

Davis, Turimumahoro [11] conducted a household randomised trial in Uganda to evaluate the impact of SMS-facilitated results delivery on completing TB evaluation. Within the trial, they used CommCare as a data collection tool but powered to capture contact investigative information in the community. CommCare was used in both the intervention and standard of care arm. However, the intervention arm participants had their sputum collected in the household, and an algorithm was applied to guide outreach workers on further evaluation steps after screening. The intervention arm participants also received specific SMS in their preferred language with instructions to visit the clinic to complete the evaluation. Although the CommCare application worked well in collecting data in both arms, the SMS-facilitated intervention did not improve the completion of TB evaluation in the intervention arm compared to the standard of care. There were also no differences in microbiologically and clinically confirmed TB between the arms. The low proportion of contacts who could produce sputum in the households was identified as one of the reasons why the intervention may not have worked. In addition, there were major challenges with the SMS system, such as only 20% of texts sent out being successfully delivered to the intended contacts. Also, the design of the contact tracing application and the logic required to send correct SMSs on time were complex. Therefore, the authors suggested using a design thinking approach to develop appropriate technologies for contact tracing to be successful [35]. A sub-study within the same trial also evaluated other technologies that could uniquely improve contact tracing, such as the fingerprint to identify contacts (Armstrong-Hough et al., 2017). Fingerprinting was found to be feasible, but its acceptability was modest. Adoption of the fingerprint technology was also suboptimal among the users [36].

### USSD: Tambua-TB [13]

In Tanzania, the Challenge TB consortium funded by the USAID helped The National Tuberculosis and Leprosy Control Programme (NTLP) develop the Tambua TB application for contacts` self-screening using a USSD to increase knowledge of TB using messaging services [13]. The development of the application took over a year until its first deployment in 2018. Public health experts, developers and government officials conducted workshops across all administrative levels to brainstorm, develop content for the application, ensure compliance with national guidelines and pilot the application. Messages were also built into the App, targeting symptomatic and asymptomatic contacts. Ultimately, the USSD application could support three modules; TB screening to the general population, linkage to care through automated referrals for the symptomatic, and messages of newly diagnosed patients to support treatment. The government participated in all the stages; at the App launch in 2018, other regions where the Challenge TB programme was not present also got to use the Tambua TB with government support. In addition, there was the extensive promotion of the application through multimedia. Within 8 months of implementation from launch, more than 229 000 people had self-screened and at least 166 000 were reportedly presumptive. The main challenge experienced was that not all were truly presumptive, as some used self-screening applications for experimenting or out of curiosity. No further information is available on the use or evaluation of the App; however, future updates were planned to use the linkage, contact tracing and referral features for the presumptive. This is envisaged to improve the genuinely presumptive numbers. Again, as seen in AfyaData (IssueLab, 2017, Karimuribo et al., 2017), government involvement and multi-stakeholder involvement proved to be a major requisite for successful implementation.

### Kobo: Wetaase COVID-19 App [15]

The Wetaase App was developed and piloted in Uganda in 2021 to assess its feasibility in increasing alerts for COVID-19. Kobo is open-source software that works like ODK and CommCare, allowing for the development of data collection tools, management, and data visualisation. KOBO is supported by large organisations such as USAID, CISCO, and the World Bank. In addition, its development is supported by the Harvard Humanitarian Initiative and Brigham and Women’s Hospital.

The Wetaase App was designed to be used by the contacts to report their symptoms daily. Only thirty households were selected purposively and included in the study. Trained research assistants helped one household member install the application on a smartphone and taught them how to report daily symptoms for three months. Other household members would write their symptoms and be captured on the App, uniquely identifying their names. Data were monitored daily to ensure that all household members were completing. Qualitative data were also collected to understand if users liked the concept of using the App, if it was easy and robust, if they were willing to pay for the costs of data and if they would recommend it to others. Only 101 contacts were enrolled; over the 90-day follow-up period, there was a 78% completion rate. Out of those that completed the questions on the App, only 0.86% reported having at least one symptom. None of the participants reported contact with a suspected or confirmed COVID-19 case. Participants liked using the App, and it also was intuitive. However, the sustainability of the App is unclear in the absence of the high COVID-19 numbers. The authors attributed the failure to find any COVID-19 to these dwindling numbers at the implementation time. No technical challenges on the App were reported.

**Ebola syndromic surveillance using calls and alerts [2]**

Jia and Mohamed (2024) evaluated the use of cell phone messaging for Ebola syndromic surveillance in high-risk settings in Southern Sierra Leone. The study aimed to assess the effectiveness of text messages and voice calls for community-based surveillance of Ebola hemorrhagic fever (EHF). The Moyamba District Health Management Team used cell phones to send and receive alert messages, including SMS texts and voice calls, reporting suspect and confirmed EHF cases. Community members with cell phones reported suspected cases and mortalities to the Moyamba District Health Management Team. Upon receiving these alerts, the team promptly followed up, often within 24 hours. The district was divided into three zones, each managed by an Ebola syndromic surveillance zonal commander. These commanders coordinated the response efforts, including evacuating suspected cases to treatment centres and dispatching burial teams for deceased cases.

Results indicated that cell phone alerts were effective in reporting EHF cases and mortalities, suggesting that traditional surveillance methods underreported cases. A strong correlation existed between cell phone-reported suspect cases and deaths, highlighting the potential of this technology for timely and accurate surveillance.

In October 2014, the Moyamba District Health Management Team recorded 129 suspected Ebola hemorrhagic fever (EHF) cases and 199 mortalities using a cell phone-based community syndromic surveillance system. Out of 260 alerts, 85.77% (223) were followed up within 24 hours, 13.85% were unmet, and 0.38% were false. This system proved more effective and timely in reporting compared to traditional sentinel surveillance, which recorded only 50 confirmed cases and 4 mortalities between August and September 2014 with weak correlations between suspect cases and deaths.

The study concluded that cell phone technology could be a valuable tool for epidemic surveillance in resource-poor settings, offering scalability and cost-effectiveness for monitoring large populations. The use of cell phones significantly enhanced the accuracy and speed of Ebola case reporting and mortality surveillance. However, it emphasised the need for future research to validate data accuracy and address issues such as unmet and false alerts.

**Contact tracing using cellphone towers and subpoenas [4]**

Wolfe et al. (2015) assessed the contact tracing activities during the Ebola Virus Disease (EVD) outbreak in the Duport Road area of Monrovia, Liberia, in November 2015. This study highlighted innovative methods, including the covert use of cellphone tower technology and subpoenas, to locate missing contacts of confirmed EVD cases.

During the outbreak, contact tracing teams faced challenges in identifying and monitoring contacts due to some individuals fleeing or hiding due to fear and stigma. To address this, the Ministry of Health subpoenaed mobile phone companies to access phone records of missing contacts. By analysing these records, the teams determined the locations where calls were made or received and conducted house-to-house searches in those areas. The use of cellphone tower technology proved effective in identifying the whereabouts of missing contacts.

About 29 contacts who were missing at the start of the study were found using phone records and other methods employed in the study. while integrating cellphone tower technology and legal measures like subpoenas significantly enhanced the contact tracing process, the study was marred with challenges of breaches of privacy and stigmatisation.

**The 117 call alert system in Sierra Leone [5]**

The Government of Sierra Leone repurposed a national toll-free hotline, 117, in August 2014 to quickly identify, investigate, isolate, and test potential Ebola cases and deaths. Community members used the system to report sick individuals and deaths from any cause, allowing District Health Management Teams to respond swiftly. These teams investigated live alerts and conducted Ebola testing, while all deaths received safe and dignified burials according to a standard protocol.

Between September 2014 and December 2016, about 350000 complete non-prank calls were made to the 117 system. The peak daily death alerts reached 9,344 in October 2014, while live alerts peaked at 3,031 in December 2014. Both types of alerts decreased as Ebola cases declined nationwide and continued to drop post-epidemic. Death alerts consistently outnumbered live alerts, indicating potential missed opportunities for identifying and referring suspected Ebola cases. Death reports continued through the 117 system after the peak of new Ebola cases but reports rapidly declined across all regions after the epidemic ended in November 2015. Live alerts also rapidly reduced immediately after the peak of weekly new Ebola cases and even more so after the epidemic ended. However, a positive indicator was that the 117 system also received calls for non-Ebola-related issues such as water ruptures, perinatal emergencies and motor vehicle accidents and questions about the lifting of Ebola-related restrictions. Since August 2016, the 117 system has continued capturing basic data for the cause and place of death. The authors contend that with adequate political support, funding, and ongoing community engagement, the 117 system could serve as a model for toll-free, phone-based death reporting in other low- and middle-income countries[5].

**TB HealthCheck and COVID-alert app [18]**

During the COVID-19 outbreak in 2020, the Department of Health in South Africa partnered with Reach Digital Health (formerly the Praekelt Foundation) to develop HealthConnect, a suite of digital tools for managing the pandemic [37, 38]. This suite included COVIDAlert, HealthCheck, and HealthWorkerAlert. COVIDAlert provided on-demand information on COVID-19 case numbers and best practices to the general public and it reached over 8 million users in South Africa by the end of 2020 [37]. HealthCheck was designed for contact tracing and case finding by enabling users to check their symptoms, assess their COVID-19 risk, and get COVID-19 screening clearance. At least 11 million screenings were done using the HealthCheck app in 2021. The app found that 94% (9.4 million of 10 million) screenings were low risk and only 0.05% (50000/10000000) were high risk, thus helping limit contact with high-risk individuals [37].

Building on the success of the COVID-19 tools, the National Department of Health (NDoH), in partnership with the Clinton Health Access Initiative, Reach Digital Health and Made2Fly Creative, launched TB HealthCheck on 19 March 2021 [39]. This was also designed as a WhatsApp and USSD-based self-screening app [18]. TB HealthCheck was accessible via WhatsApp by sending *TB* to +27 60 012 3456 or through USSD by dialling *134*832*5#[40]. The app guided users through a series of questions about symptoms and provided testing recommendations based on their responses. However, detailed statistical results regarding the long-term impact and outcomes of the TB HealthCheck app beyond the initial implementation phase are not widely available and an evaluation is not sufficiently done [37]. The app was integrated into the broader HealthCheck system, which also covered COVID-19 risk assessments, ensuring a comprehensive digital health tool for the public. The success of HealthConnect was attributed to several factors, including its adaptability, the strong partnerships between the government and tech developers, and the comprehensive outreach campaigns that ensured widespread adoption[38].

**References**

1. Sacks JA, Zehe E, Redick C, Bah A, Cowger K, Camara M, et al. Introduction of mobile health tools to support ebola surveillance and contact tracing in guinea. Glob Health Sci Pract. 2015;3(4):646-59. PMID:26681710. doi:10.9745/GHSP-D-15-00207.

2. Jia K, Mohamed K. Evaluating the use of cell phone messaging for community ebola syndromic surveillance in high risked settings in southern sierra leone. African Health Sciences. 2015;15(3):797-802. PMID:26957967. doi:10.4314/ahs.v15i3.13.

3. Tom-Aba D, Olaleye A, Olayinka AT, Nguku P, Waziri N, Adewuyi P, et al. Innovative technological approach to ebola virus disease outbreak response in nigeria using the open data kit and form hub technology. PLoS One. 2015;10(6):e0131000. PMID:26115402. doi:10.1371/journal.pone.0131000.

4. Wolfe CM, Hamblion EL, Schulte J, Williams P, Koryon A, Enders J, et al. Ebola virus disease contact tracing activities, lessons learned and best practices during the duport road outbreak in monrovia, liberia, november 2015. PLoS Neglected Tropical Diseases. 2017;11(6):e0005597. PMID:28575034. doi:10.1371/journal.pntd.0005597.

5. Alpren C, Jalloh MF, Kaiser R, Diop M, Kargbo S, Castle E, et al. The 117 call alert system in sierra leone: From rapid ebola notification to routine death reporting. BMJ Global Health. 2017;2(3):e000392. PMID:28948044. doi:10.1136/bmjgh-2017-000392.

6. Adeoye OO, Tom-Aba D, Ameh CA, Ojo OE, Ilori EA, Gidado SO, et al. Implementing surveillance and outbreak response management and analysis system (sormas) for public health in west africa-lessons learnt and future direction. International Journal of Tropical Disease & Health. 2017;22(2):1-17. doi:10.9734/IJTDH/2017/31584.

7. Danquah LO, Hasham N, MacFarlane M, Conteh FE, Momoh F, Tedesco AA, et al. Use of a mobile application for ebola contact tracing and monitoring in northern sierra leone: A proof-of-concept study. BMC Infect Dis. 2019;19(1):810. PMID:31533659. doi:10.1186/s12879-019-4354-z.

8. Whitesell A, Bustamante ND, Stewart M, Freeman J, Dismer AM, Alarcon W, et al. Development and implementation of the ebola exposure window calculator: A tool for ebola virus disease outbreak field investigations. PLoS One. 2021;16(8):e0255631. PMID:34352008. doi:10.1371/journal.pone.0255631.

9. Chisunkha B, Banda H, Thomson R, Squire SB, Mortimer K. Implementation of digital technology solutions for a lung health trial in rural malawi. Eur Respir J. 2016;47(6):1876-9. PMID:27076597. doi:10.1183/13993003.00045-2016.

10. Ha YP, Tesfalul MA, Littman-Quinn R, Antwi C, Green RS, Mapila TO, et al. Evaluation of a mobile health approach to tuberculosis contact tracing in botswana. J Health Commun. 2016;21(10):1115-21. PMID:27668973. doi:10.1080/10810730.2016.1222035.

11. Davis JL, Turimumahoro P, Meyer AJ, Ayakaka I, Ochom E, Ggita J, et al. Home-based tuberculosis contact investigation in uganda: A household randomised trial. ERJ Open Research. 2019;5(3). PMID:31367636. doi:10.1183/23120541.00112-2019.

12. Szkwarko D, Amisi JA, Peterson D, Burudi S, Angala P, Carter EJ. Using a mobile application to improve pediatric presumptive tb identification in western kenya. Int J Tuberc Lung Dis. 2021;25(6):468-74. PMID:34049609. doi:10.5588/ijtld.20.0890.

13. Diaz N, Moturi E. Using mhealth to self-screen and promote tb awareness in tanzania. Tanzania: Challenge TB; 2019.

14. URC. Usaid/south africa tuberculosis south africa project (tbsap) midterm evaluation report. 2020.

15. Mugenyi L, Nsubuga RN, Wanyana I, Muttamba W, Tumwesigye NM, Nsubuga SH. Feasibility of using a mobile app to monitor and report covid-19 related symptoms and people's movements in uganda. PLoS One. 2021;16(11):e0260269. PMID:34797878. doi:10.1371/journal.pone.0260269.

16. Owoyemi A, Ikpe R, Toye M, Rewane A, Abdullateef M, Obaseki E, et al. Mobile health approaches to disease surveillance in africa; wellvis covid triage tool. Digital Health. 2021;7:2055207621996876. PMID:33680485. doi:10.1177/2055207621996876.

17. Rajput ZA, Mbugua S, Amadi D, Chepngeno V, Saleem JJ, Anokwa Y, et al. Evaluation of an android-based mhealth system for population surveillance in developing countries. Journal of the American Medical Informatics Association : JAMIA. 2012;19(4):655-9. PMID:22366295. doi:10.1136/amiajnl-2011-000476.

18. Praekelt.org. Tb healthcheck puts tuberculosis self-screening in everyone’s hands ahead of world tb day: Praekelt.org; 2021 [cited 2023 May 8, 2023]. Available from: <https://www.praekelt.org/news>.

19. Karimuribo ED, Mutagahywa E, Sindato C, Mboera L, Mwabukusi M, Kariuki Njenga M, et al. A smartphone app (afyadata) for innovative one health disease surveillance from community to national levels in africa: Intervention in disease surveillance. JMIR Public Health and Surveillance. 2017;3(4):e94. PMID:29254916. doi:10.2196/publichealth.7373.

20. Grainger C. A software for disease surveillance and outbreak response-insights from implementing sormas in nigeria and ghana. Germany: Federal Ministry for Economic Cooperation and Development (BMZ). 2020.

21. Chikwe I, Lois O. Sormas in nigeria: Adapting a fully integrated surveillance system to track covid-19: Exemplars in Global Health; 2022 [cited 2022. Available from: <https://www.exemplars.health/emerging-topics/epidemic-preparedness-and-response/digital-health-tools/sormas-nigeria>.

22. Fahnrich C, Denecke K, Adeoye OO, Benzler J, Claus H, Kirchner G, et al. Surveillance and outbreak response management system (sormas) to support the control of the ebola virus disease outbreak in west africa. Eurosurveillance. 2015;20(12). PMID:25846493. doi:10.2807/1560-7917.es2015.20.12.21071.

23. Gaythorpe K, Morris A, Imai N, Stewart M, Freeman J, Choi M. Chainchecker: An application to visualise and explore transmission chains for ebola virus disease. PLoS One. 2021;16(2):e0247002. PMID:33606709. doi:10.1371/journal.pone.0247002.

24. Banda HT, Mortimer K, Bello GA, Mbera GB, Namakhoma I, Thomson R, et al. Informal health provider and practical approach to lung health interventions to improve the detection of chronic airways disease and tuberculosis at primary care level in malawi: Study protocol for a randomised controlled trial. Trials. 2015;16(1):1-11.

25. Littman-Quinn R, Chandra A, Schwartz A, Fadlelmola FM, Ghose S, Luberti AA, et al. Mhealth applications for telemedicine and public health intervention in botswana. IST-Africa Conf Proc, IST. 2011.

26. Mosweunyane G, Nkgau T, Makhura O, Seipone T, editors. Ussd system for tb contact tracing: Server, database and security issues. 9th European Conference on IS Management and Evaluation, ECIME 2015; 2015: Academic Conferences and Publishing International Limited.

27. Ncube B, Mars M, Scott RE. The need for a telemedicine strategy for botswana? A scoping review and situational assessment. BMC Health Services Research. 2020;20(1):794. PMID:32843017. doi:10.1186/s12913-020-05653-0.

28. Wools-Kaloustian KK, Sidle JE, Selke HM, Vedanthan R, Kemboi EK, Boit LJ, et al. A model for extending antiretroviral care beyond the rural health centre. Journal of the International AIDS Society. 2009;12(1):22. PMID:19788755. doi:10.1186/1758-2652-12-22.

29. Anokwa Y, Ribeka N, Parikh T, Borriello G, Were MC, editors. Design of a phone-based clinical decision support system for resource-limited settings. 5th International Conference on Information and Communication Technologies and Development, ICTD 2012; 2012; Atlanta, GA.10.1145/2160673.2160676.

30. Were MC, Kariuki J, Chepng'eno V, Wandabwa M, Ndege S, Braitstein P, et al. Leapfrogging paper-based records using handheld technology: Experience from western kenya. Medinfo 2010: IOS Press; 2010. p. 525-9.

31. Divi N, Smolinski M. Epihacks, a process for technologists and health experts to cocreate optimal solutions for disease prevention and control: User-centered design approach. J Med Internet Res. 2021;23(12):e34286. PMID:34807832. doi:10.2196/34286.

32. IssueLab. The dodres project: Revolutionizing human and animal disease surveillance in tanzania2017 June 20, 2022 [cited 2022 June 20, 2022]. Available from: <https://search.issuelab.org/resource/the-dodres-project-revolutionizing-human-and-animal-disease-surveillance-in-tanzania.html>.

33. Gates B. Meet the heroes in africa fighting back against this pandemic—and working to prevent the next one2021 June 20, 2022 [cited 2022 June 20, 2022]. Available from: <https://www.gatesnotes.com/Health/These-African-heroes-are-fighting-back-against-the-pandemic>.

34. Brunette W. Open data kit 2: Building mobile application frameworks for disconnected data management: University of Washington; 2020.9798684670749.

35. Meyer AJ, Armstrong-Hough M, Babirye D, Mark D, Turimumahoro P, Ayakaka I, et al. Implementing mhealth interventions in a resource-constrained setting: Case study from uganda. JMIR Mhealth Uhealth. 2020;8(7):e19552. PMID:32673262. doi:10.2196/19552.

36. White EB, Meyer AJ, Ggita JM, Babirye D, Mark D, Ayakaka I, et al. Feasibility, acceptability, and adoption of digital fingerprinting during contact investigation for tuberculosis in kampala, uganda: A parallel-convergent mixed-methods analysis. J Med Internet Res. 2018;20(11):e11541. PMID:30442637. doi:10.2196/11541.

37. Ramneek A, Vincent Z, Gaurang T, Gustav P, Debbie R. Healthconnect in south africa: A chatbot tool for pandemic response: Exemplars in Global Health; 2021 [Available from: <https://www.exemplars.health/emerging-topics/epidemic-preparedness-and-response/digital-health-tools/healthconnect-in-south-africa%E2%80%AF?login=true>.

38. Global Innovation Fund. Completion assessment executive summary : Healthconnect 2022 [cited 2024 July 26, 2024]. Available from: <https://www.globalinnovation.fund/assets/uploads/PDF-Documents/Completion-Assessment/HealthConnect-Investment-Assessment-Executive-Summary.pdf>.

39. Department of Education North West Government. National department of health and clinton health access initiative: Department of Education North West Government,; 2023 [Available from: <https://desd.nwpg.gov.za/?news=national-department-of-health-and-clinton-health-access-initiative>.

40. Department of Health South Africa. Mkhize highlights the way forward for sa’s tb response: Department of Health South Africa,; 2021 [Available from: <https://sacoronavirus.co.za/2021/02/05/mkhize-highlights-the-way-forward-for-sas-tb-response/>.
