# Supplementary material for: Using mHealth Technologies for Case Finding in Tuberculosis and Other Infectious Diseases in Africa: Systematic Review
Source: JMIR Mhealth Uhealth. 2024 Aug 26;12:e53211. doi: 10.2196/53211 (PMC11384173; doi:10.2196/53211)
Supplement: Multimedia Appendix 5 [file mhealth_v12i1e53211_app5.pdf]

PRISMA 2020 Checklist

Section and Topic	Item #	Checklist item	Location where item is reported
**TITLE**			
Title	1	Identify the report as a systematic review.	Title: this is the title below<br>USING MHEALTH TECHNOLOGIES TO FIND MISSING INFECTIOUS DISEASES IN AFRICA: **A SYSTEMATIC REVIEW**
**ABSTRACT**			
Abstract	2	See the PRISMA 2020 for Abstracts checklist.	Abstract
**INTRODUCTION**			
Rationale	3	Describe the rationale for the review in the context of existing knowledge.	Abstract and Introduction
Objectives	4	Provide an explicit statement of the objective(s) or question(s) the review addresses.	Introduction
**METHODS**			
Eligibility criteria	5	Specify the inclusion and exclusion criteria for the review and how studies were grouped for the syntheses.	Methods under Inclusion and exclusion criteria subsection
Information sources	6	Specify all databases, registers, websites, organisations, reference lists and other sources searched or consulted to identify studies. Specify the date when each source was last searched or consulted.	Specified under literature search including dates
Search strategy	7	Present the full search strategies for all databases, registers and websites, including any filters and limits used.	Attached as an appendix “search terms”
Selection process	8	Specify the methods used to decide whether a study met the inclusion criteria of the review, including how many reviewers screened each record and each report retrieved, whether they worked independently, and if applicable, details of automation tools used in the process.	Included under screening literature, extracting and analysis. Methods for inclusion partially included in the inclusion and exclusion criteria but completed in the subsequent screening literature, extracting and analysis section
Data collection process	9	Specify the methods used to collect data from reports, including how many reviewers collected data from each report, whether they worked independently, any processes for obtaining or confirming data from study investigators, and if applicable, details of automation tools used in the process.	Under Screening literature, extracting and analysis
Data items	10a	List and define all outcomes for which data were sought. Specify whether all results that were compatible with each outcome domain in each study were sought (e.g. for all measures, time points, analyses), and if not, the methods used to decide which results to collect.	Under Screening literature, extracting and analysis

PRISMA 2020 Checklist

Section and Topic	Item #	Checklist item	Location where item is reported
	10b	List and define all other variables for which data were sought (e.g. participant and intervention characteristics, funding sources). Describe any assumptions made about any missing or unclear information.	Under Screening literature, extracting and analysis
Study risk of bias assessment	11	Specify the methods used to assess risk of bias in the included studies, including details of the tool(s) used, how many reviewers assessed each study and whether they worked independently, and if applicable, details of automation tools used in the process.	Quality assessments and risk of bias
Effect measures	12	Specify for each outcome the effect measure(s) (e.g. risk ratio, mean difference) used in the synthesis or presentation of results.	N/A
NSynthesis methods	13a	Describe the processes used to decide which studies were eligible for each synthesis (e.g. tabulating the study intervention characteristics and comparing against the planned groups for each synthesis (item #5)).	Included under screening literature, extracting and analysis. Methods for inclusion partially included in the inclusion and exclusion criteria but completed in the subsequent screening literature, extracting and analysis section
	13b	Describe any methods required to prepare the data for presentation or synthesis, such as handling of missing summary statistics, or data conversions.	Screening literature, extracting and analysis
	13c	Describe any methods used to tabulate or visually display results of individual studies and syntheses.	Screening literature, extracting and analysis
	13d	Describe any methods used to synthesize results and provide a rationale for the choice(s). If meta-analysis was performed, describe the model(s), method(s) to identify the presence and extent of statistical heterogeneity, and software package(s) used.	Screening literature, extracting and analysis
	13e	Describe any methods used to explore possible causes of heterogeneity among study results (e.g. subgroup analysis, meta-regression).	N/A
	13f	Describe any sensitivity analyses conducted to assess robustness of the synthesized results.	N/A
Reporting bias assessment	14	Describe any methods used to assess risk of bias due to missing results in a synthesis (arising from reporting biases).	N/A
Certainty assessment	15	Describe any methods used to assess certainty (or confidence) in the body of evidence for an outcome.	N/A
**RESULTS**			
Study selection	16a	Describe the results of the search and selection process, from the number of records identified in the search to the number of studies included in the review, ideally using a flow diagram.	Results, subsection called literature search and in table 1
	16b	Cite studies that might appear to meet the inclusion criteria, but which were excluded, and explain why they were excluded.	listed in the appendices as well as the reason for the exclusion
Study characteristics	17	Cite each included study and present its characteristics.	Results, under the description of the studies
Risk of bias in	18	Present assessments of risk of bias for each included study.	Attached as an

PRISMA 2020 Checklist

Section and Topic	Item #	Checklist item	Location where item is reported
studies			appendix/supplementary information
Results of individual studies	19	For all outcomes, present, for each study: (a) summary statistics for each group (where appropriate) and (b) an effect estimate and its precision (e.g. confidence/credible interval), ideally using structured tables or plots.	Results, in a section called “Effectiveness of the mHealth technologies and reporting of outcomes” and in table 2
Results of syntheses	20a	For each synthesis, briefly summarise the characteristics and risk of bias among contributing studies.	They were summarised in the provided supplementary text
	20b	Present results of all statistical syntheses conducted. If meta-analysis was done, present for each the summary estimate and its precision (e.g. confidence/credible interval) and measures of statistical heterogeneity. If comparing groups, describe the direction of the effect.	N/A
	20c	Present results of all investigations of possible causes of heterogeneity among study results.	N/A
	20d	Present results of all sensitivity analyses conducted to assess the robustness of the synthesized results.	N/A
Reporting biases	21	Present assessments of risk of bias due to missing results (arising from reporting biases) for each synthesis assessed.	N/A
Certainty of evidence	22	Present assessments of certainty (or confidence) in the body of evidence for each outcome assessed.	N/A
**DISCUSSION**			
Discussion	23a	Provide a general interpretation of the results in the context of other evidence.	In the discussion, the first 2 paragraphs
	23b	Discuss any limitations of the evidence included in the review.	Limitations section under the discussion
	23c	Discuss any limitations of the review processes used.	Not discussed
	23d	Discuss implications of the results for practice, policy, and future research.	Recommendations section under the discussion
**OTHER INFORMATION**			
Registration and protocol	24a	Provide registration information for the review, including register name and registration number, or state that the review was not registered.	N/A
	24b	Indicate where the review protocol can be accessed, or state that a protocol was not prepared.	Protocol was not prepared
	24c	Describe and explain any amendments to information provided at registration or in the protocol.	N/A
Support	25	Describe sources of financial or non-financial support for the review, and the role of the funders or sponsors in the review.	PhD supervisors, colleagues and fellow professionals in the TB contact tracing field. They are all co-authors
Competing	26	Declare any competing interests of review authors.	None

PRISMA 2020 Checklist

Section and Topic	Item #	Checklist item	Location where item is reported
interests			
Availability of data, code and other materials	27	Report which of the following are publicly available and where they can be found: template data collection forms; data extracted from included studies; data used for all analyses; analytic code; any other materials used in the review.	The following are in the supplementary information;<br>Data extraction form<br>Search strategy<br>Risk of bias tables

From: Page MJ, McKenzie JE, Bossuyt PM, Boutron I, Hoffmann TC, Mulrow CD, et al. The PRISMA 2020 statement: an updated guideline for reporting systematic reviews. BMJ 2021;372:n71. doi: 10.1136/bmj.n71
For more information, visit: http://www.prisma-statement.org/
